# GKRP-dependent modulation of feeding behavior by tanycyte-released monocarboxylates

**DOI:** 10.7150/thno.66634

**Published:** 2022-01-03

**Authors:** Magdiel Salgado, Roberto Elizondo-Vega, Pablo S Villar, Macarena Konar, Scarlet Gallegos, Estefanía Tarifeño-Saldivia, Patricia Luz-Crawford, Luis G. Aguayo, Ricardo C. Araneda, Elena Uribe, María Ángeles García-Robles

**Affiliations:** 1 Departamento de Biología Celular, Facultad de Ciencias Biológicas, Universidad de Concepción, Chile.; 2 Department of Biology, University of Maryland, USA.; 3 Departamento de Bioquímica y Biología Molecular, Facultad de Ciencias Biológicas, Universidad de Concepción, Concepción, Chile.; 4 Centro de Investigación Biomédica, Facultad de Medicina, Universidad de Los Andes, Santiago, Chile.; 5 Departamento de Fisiología, Facultad de Ciencias Biológicas, Universidad de Concepción, Chile.

**Keywords:** Tanycyte, GKRP, lactate, β-hydroxybutyrate, feeding behavior, obesity

## Abstract

**Objectives**: Glucokinase Regulatory Protein (GKRP) is the only known endogenous modulator of glucokinase (GK) localization and activity to date, and both proteins are localized in tanycytes, radial glia-like cells involved in metabolic and endocrine functions in the hypothalamus. However, the role of tanycytic GKRP and its impact on the regulation of feeding behavior has not been investigated. Here, we hypothesize that GKRP regulates feeding behavior by modulating tanycyte-neuron metabolic communication in the arcuate nucleus.

**Methods**: We used primary cultures of tanycytes to evaluate the production of lactate and β-hydroxybutyrate (βHB). Similarly, we examined the electrophysiological responses to these metabolites in pro-opiomelanocortin (POMC) neurons in hypothalamic slices. To evaluate the role of GKRP in feeding behavior, we generated tanycyte-selective GKRP-overexpressing and GKRP-knock down mice (GKRP^t^-OE and GKRP^t^-KD respectively) using adenovirus-mediated transduction.

**Results**: We demonstrated that lactate release induced by glucose uptake is favored in GKRP-KD tanycytes. Conversely, tanycytes overexpressing GKRP showed an increase in βHB efflux induced by low glucose concentration. In line with these findings, the excitability of POMC neurons was enhanced by lactate and decreased in the presence of βHB. In GKRP^t^-OE rats, we found an increase in post-fasting food avidity, whereas GKRP^t^-KD caused a significant decrease in feeding and body weight, which is reverted when MCT1 is silenced.

**Conclusion**: Our study highlights the role of tanycytic GKRP in metabolic regulation and positions this regulator of GK as a therapeutic target for boosting satiety in patients with obesity problems.

## Introduction

Glucokinase (GK) regulatory protein (GKRP) is a 69-kDa protein that in the liver plays a key role during hypoglycemia by inhibiting GK activity and also sequestering GK in the nucleus of hepatocytes, preventing glucose catabolism in fasting periods when hepatic glucose export occurs 1. Without this inhibition of GK, a futile glucose phosphorylation/dephosphorylation cycle would be propagated during low-energy conditions. Since GK has a key role in glucose homeostasis, this enzyme has aroused great interest as a therapeutic target against metabolic diseases which contribute to the development of obesity. For instance, GK activity might be decreased in patients with type 2 diabetes, which has led to numerous attempts to find drugs that stimulate GK activity 2. Among the candidates, GK activators have generated much interest since the 1990s as a potential new class of antidiabetic drugs. However, despite promising preliminary results, the clinical use GK activators was unsuccessful due to their adverse effects characterized by hypoglycemia 2-4. In this context, it has been proposed that the endogenous disruption of GK and GKRP interaction in the liver could lead to better results 5, with a reduction in hepatic glucose output without the risk of insulin-dependent hypoglycemia 6, 7.

Interestingly, recent work has shown that GK and GKRP are also expressed in the hypothalamus, especially in tanycytes 8, 9, a type of radial glia-like cells that cover the walls and floor of the third ventricle (3V) in the basal hypothalamus. Due to their anatomical position, tanycytes appear strategically positioned to detect changes in glucose concentration from cerebrospinal fluid (CSF) and from fenestrated vessels in the median eminence. In turn, their elongated processes project toward the arcuate nucleus (ARC), where feeding-regulating neurons reside 10, suggesting that tanycytes could convey metabolic signalling to ARC neurons. Supporting this intercellular communication, we have shown that disruption of several tanycyte proteins involved in glucose metabolism, including GLUT2 11, GK 12, and MCT1-4 13, 14, increase food intake in rats. This led us to propose that glucose from the CSF is taken by tanycytes using GLUT2, which is then metabolized to lactate with the participation of GK. Therefore, released lactate from tanycytes could act as an intercellular messenger that activates anorexigenic responses. Although it remains unknown if tanycytes play a role in communicating to ARC neurons during a low-energy state, MCT1 disruption also induced abnormal satiety in fasted rats 13. Since MCT1 also transports ketone bodies (KB), these metabolites may have a role in this state. β-hydroxybutyrate (βHB) is the main KB produced by the liver from the metabolism of fatty acids in a fasting condition. Interestingly, mouse cortical astrocytes also produce βHB using an AMPK-dependent mechanism 15, but it is unknown if a similar mechanism occurs in tanycytes.

In this study, we tested the hypothesis that GKRP modulates monocarboxylate-dependent tanycyte-neuron metabolic communication in the ARC. For this, the function of GKRP was increased and decreased using adenoviral vectors. Our results provide novel insights on the role of GKRP expression on tanycytes and further highlight the role of tanycytes in satiety regulation.

## Results

### Tanycyte production of monocarboxylates is regulated by energetic conditions

**Fig 1:**
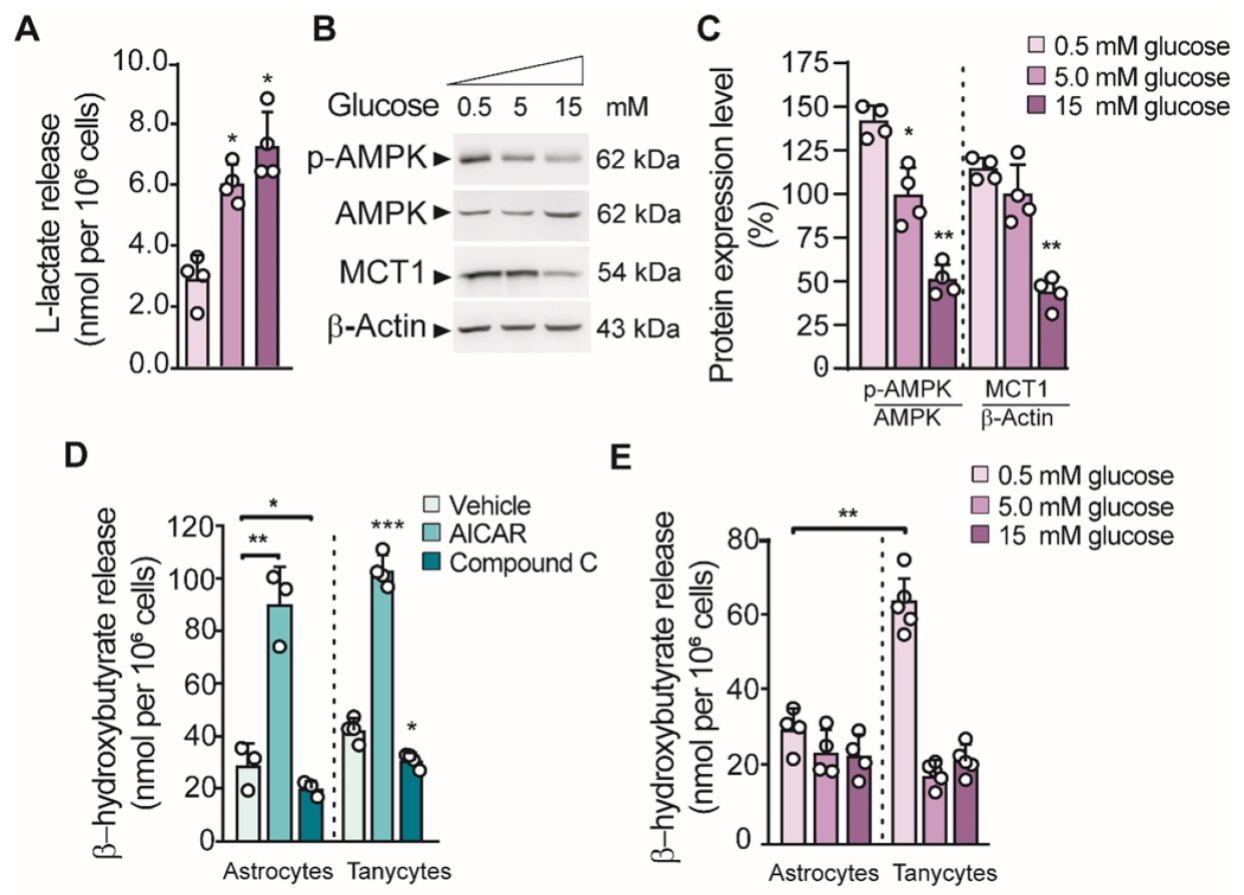
** Glial cell production of βHB and its regulation by GKRP**. **A**. Lactate production in tanycytes incubated at different glucose concentrations for 1 h. **B-C.** Western blot and quantification for phosphorylated AMPK (pAMPK), total AMPK (AMPK), MCT1 and β-actin loading control from the total protein of tanycytes after stimulating with glucose as described in A. **D**. βHB production in astrocytes and tanycytes primary cultures upon AMPK activity activation with 0.5mM Aicar or AMPK inactivation with 15µM compound C (CpC). **E**. βHB production in cortical astrocytes and tanycytes in response to different glucose concentrations for 1 h.

Previous studies showed that tanycyte cultures released L-lactate in the presence of 5 mM glucose in a time-dependent manner 16. Similarly, we found that lactate production in cultured tanycytes also increased in response to increasing concentrations of glucose (Figure 1A). Given that we detected a positive regulation of MCT1 in low glucose concentration (Figure 1B-C), we wonder if tanycytes release βHB through MCT1. In this condition, we also detected an increase in AMPK phosphorylation (p-AMPK; Thr172) in tanycytes, the key stage for inducing ketogenesis in primary astrocytes 15. Using RT-PCR, we detected in tanycytes cultures the expression of key enzymes that catalyze the βHB production (Figure S1). Furthermore, tanycytes express *Prkaa2* and* Cpt1a,* two proteins implicated in the sensing of hypoglycemia and the uptake of fatty acids by mitochondria, respectively 17. However, mRNA for 3-oxoacid CoA-transferase 1 (*Oxct1*) was not detected, which supports the possibility that βHB could be released from tanycytes. Thus, these data suggest that tanycytes express the enzymatic machinery needed to produce βHB from fatty acids.

Consistent with that described above, tanycytes produced βHB, in a mechanism that was dependent on AMPK activity (Figure 1D). Similar results were obtained using astrocyte cultures as previously reported. There was ~3-fold increase in βHB production using AICAR and a significant decrease in the presence of CpC (an AMPK agonist and antagonist, respectively) in both glial cells (Figure 1D). In contrast, βHB production was not induced by AMPK activation in unrelated cells, such as INS-1 cells (data not shown). Interstingly, the highest βHB release from tanycytes occurred at 0.5mM glucose unlike astrocytes where βHB release did not depend on glucose concentrarions (Figure 1E).

### Opposing roles of lactate and βHB on hypothalamic neuronal activity

**Fig 2:**
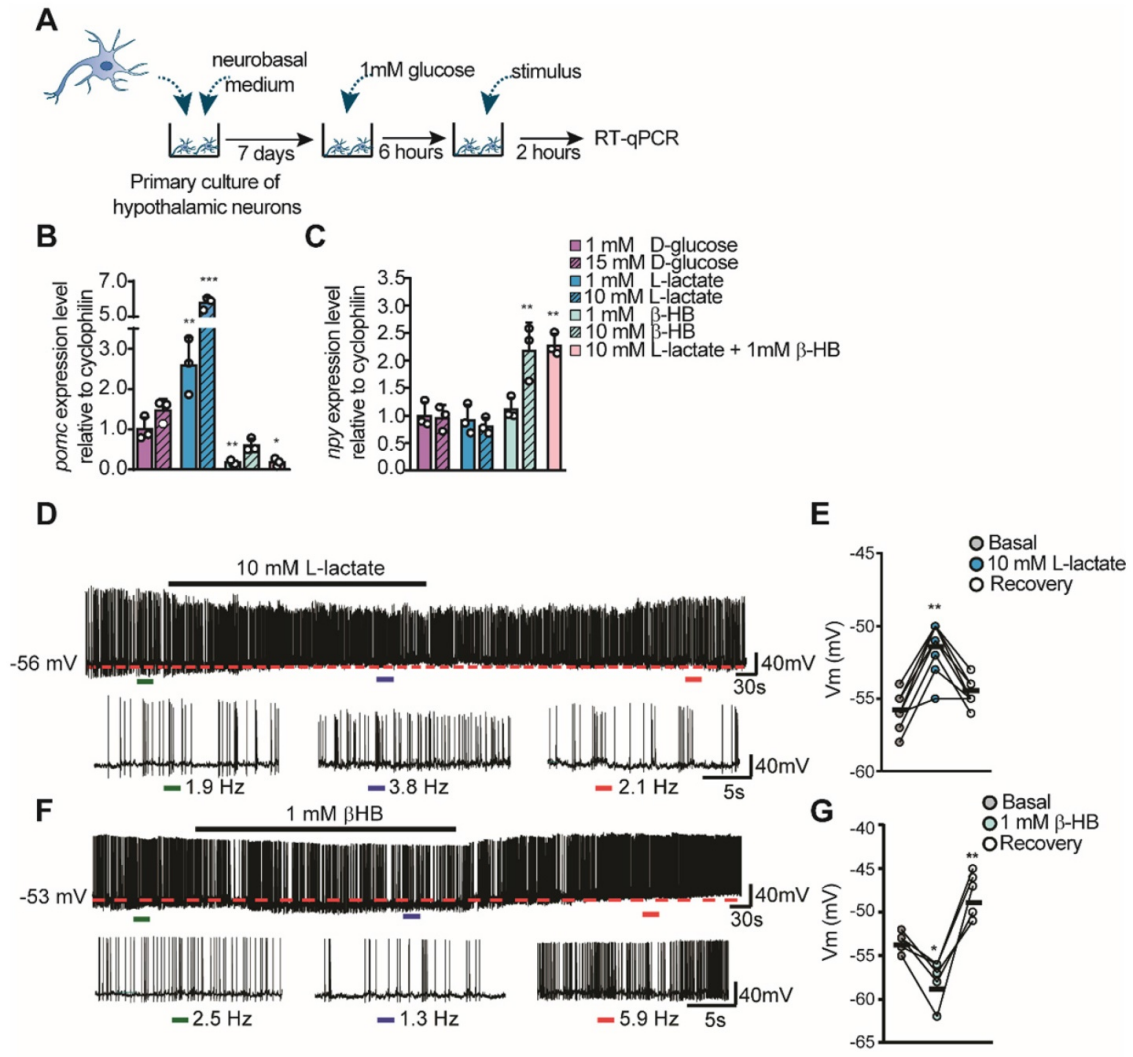
** Hypothalamic neuronal activity in response to lactate and βHB. A-C**: Hypothalamic neuronal primary cultures were processed as indicated in the temporal scheme (**A**) with the aim to measure POMC (**B**) and NPY (**C**) neuropeptide expression in response to glucose (magenta bars), L-lactate (blue bars), βHB (green bars), and a mixture of 10mM lactate and 1mM βHB (pink bars) by RT-qPCR after 2 h stimulus application. **D-E**. Current clamp recordings of mice POMC neurons in response to 10mM and in response to 1 mM lactate (**F-G**). In E and G, ΔV_m_ represents changes in membrane potential. In each recording, basal and recovery conditions correspond to aCSF with 5mM glucose. The dashed red line in each trace represents initial resting potential. Below each recording an expanded trace indicates the conditions before (green bar), during (blue bar), and after (red bar) the stimulus; with the corresponding values of firing frequency. * p < 0.05, ** p < 0.01, *** p < 0.001.

To evaluate the role of lactate and βHB over hypothalamic neuronal activity, we measured the expression of feeding-controlling neuropeptides in hypothalamic cultures containing mainly neurons by RT-qPCR (Figure 2A), as prevoulsy done 11, 14, 18. These cultures were maintained in 1 mM glucose and then we measured changes in neuropeptide mRNA levels 2 h post-stimulation as a consequence of neuropeptides released in response to different concentrations of glucose, lactate, and βHB. Interestingly, *Pomc* or *Npy* expression was not affected by stimulation with 15 mM glucose, suggesting that these cells do not respond to glucose in the absence of the normally present glial cells (Figure 2B-C, magenta bars). However, neurons incubated with L-lactate showed a robust increase in *Pomc* levels in a concentration-dependent manner (Figure 2B), while *Npy* levels were not affected by lactate at these concentrations (Figure 2C). Interestingly, only 1 mM βHB induced a decrease in *Pomc* expression of 86% compared to the basal condition (1 mM glucose). βHB evoked a 2-fold increase in Npy expression only at 10 mM βHB (Figure 2B-C, green bars); unexpectedly, 10 mM lactate and 1 mM βHB produced the same results (Figure 2C, pink bar). Similarly, neurons incubated with this mixture exhibited a significant reduction in POMC mRNA (1.0±0.3 vs. 0.2±0.1, *p =* 0.05), suggesting that the signaling mediated by βHB is more powerful and/or faster than that induced by lactate.

Next, we wanted to examine if lactate and βHB were able to alter neuronal activity in POMC neurons that are known to be important in feeding behaviors and that might help to explain our previous results. Using electrophysiological recordings from transgenic mice that express EGFP under the control of the POMC promoter, we determined the effects of lactate and βHB on neuronal firing held at the indicated potentials (Figure 2D-G). Hypothalamic slices were perfused with an external solution containing 5 mM glucose, and POMC-GPP neurons were maintained close to the threshold. Under these conditions, these POMC neurons showed abundant spiking activity with a mean frequency of 2.4 Hz ± 0.6 under control conditions (n = 24) at rest. Perfusion with 10 mM L-lactate produced a slight and reversible depolarization in the majority of POMC neurons recorded (n = 7/8), which further exacerbated the firing (shown in the expanded traces, Figure 2D). Resting values returned during the washout period. The average change in V_m_ during lactate application was 4.4 ± 1.5 mV from the control value (dotted red line in Figure 2D-E). In contrast, the administration of 1 mM βHB appeared to produce a more complex response. As shown in Figure 2F-G, perfusion of βHB produced a decrease in the spiking frequency, which was accompanied by a hyperpolarization in the resting potential (n = 5/5) (mean ΔV_m_ of -5.0 ± 1.2 mV from the control value). Interestingly, during the washout period, the value of the resting potential rebounded towards higher values in all POMC neurons recorded (mean ΔV_m_ of 4.8 ± 2.2 mV, Figure 2G), with a parallel increase in spiking. These results show a concordance between the changes in neuronal neuropeptide expression and activity of POMC neurons, indicating an opposing modulation of lactate and βHB in neuronal activity.

### Intracerebroventricular (ICV) administration of βHB induces short-term hunger

**Fig 3:**
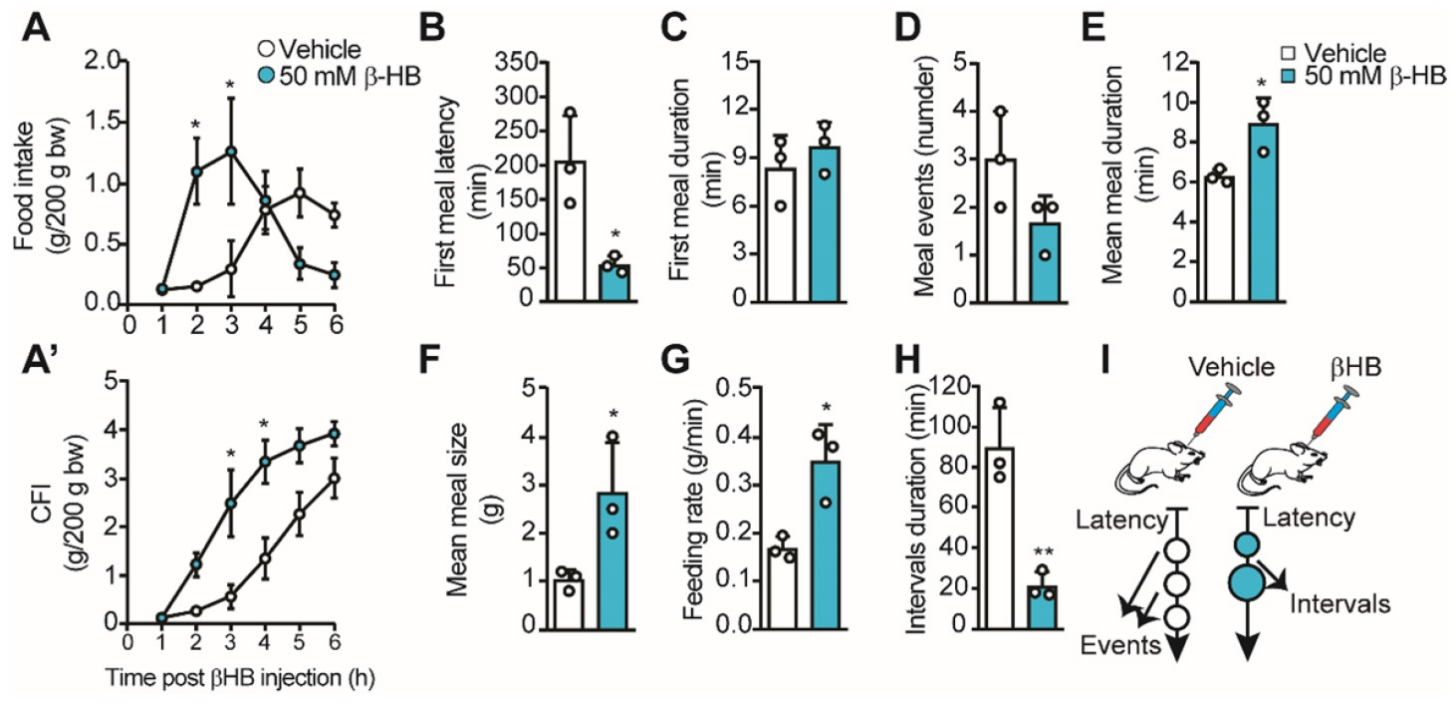
** Feeding behavior of rats upon icv βHB injection.** Adult rats were icv-injected with 10 μL of vehicle (DMSO) or βHB and then their feeding behavior was monitored.** A.** Food intake during 6h following icv injection of vehicle or βHB. **A'.** Cumulative food intake (CFI) after each injection. **B-H**. From automatic monitoring of eating behavior, we determined the latency of the first meal (**B**), the first meal duration (min, **C**), the mean number of meal events (**D**), the mean meal duration (min, **E**), the mean meal size (g, **F**), the feeding rate (g/min, **G**) and the intervals duration (min, **H**) upon βHB or vehicle injection. **I.** Schematic representation of feeding behavior of rats icv-injected with vehicle or βHB. n = 3, * p < 0.05, ** p < 0.01.

Using different approaches, we and others have proposed that the lactate released by tanycytes in response to glucose can mediate an anorexigenic response in the ARC 14, 16, 19. However, the role of tanycyte-released βHB at the hypothalamic level is unknown. Therefore, we evaluated the impact of intraventricular injections of βHB in *ad libitum* fed rats to avoid the contribution of hepatic KB. For this, we injected 10 µL of 50 mM βHB or vehicle into the 3V of pre-cannulated animals, and we recorded their food intake for 6 h, determining food consumption at each hour post βHB injection. We found a significant increase in food intake 2-3 h post βHB injection, but the intake was not different from controls after that (Figure 3A). When we plotted cumulative food intake (CFI) at the end of the 6 h, we did not detect significant differences from the control group, while a significant increase in CFI was observed between 3-4 h post-βHB injection (Figure 3A'). The early food intake in βHB treated rats could be due to higher food avidity in line with the lower latency detected at the first meal (205 ± 67 min vs. 54 ± 13 min, *p* = 0.019; Figure 3B); however, the duration of this first meal was similar to vehicle-injected rats (Figure 3C). Although the number of meal events was not significantly altered over this 6-h period, a trend towards a decrease in the events was observed in βHB-injected rats (Figure 3D, 3.0 ± 1.0 *vs.* 1.7 ± 0.6, *p* = 0.12). Interestingly, this apparent lower number in feeding events was accompanied by a longer mean duration (6.3 ± 0.3 *vs.* 8.9 ± 1.3 min, *p =* 0.026; Figure 3E), a higher average size of each meal (1.0 ± 0.2 g *vs.* 2.8±1.0 g, *p =* 0.042; Figure 3F), and a greater feeding rate (0.17 ± 0.02 *vs.* 0.35 ± 0.08 g/min, *p =* 0.017; Figure 3G) in βHB-injected rats, suggesting lower satiation post-feeding. Finally, a ˃4-fold drop in interval duration was observed upon βHB treatment (89.7 ± 19.7 min *vs.* 21.3 ± 6.6 min, *p =* 0.005; Figure 3H), suggesting lower satiety between the first and second meal event. Nevertheless, we noted that after the second feeding event, βHB-injected rats stopped eating without presenting further eating events. Therefore, icv βHB injection evokes a rapid (lower latency) and transient increase in feeding that is associated with lower satiation (higher feeding time, size, and speed) and satiety (shorter interval duration) in rats. This feeding behavior is summarized in the scheme shown in Figure 3I.

### GKRP modulates monocarboxylate release from tanycytes

**Fig 4:**
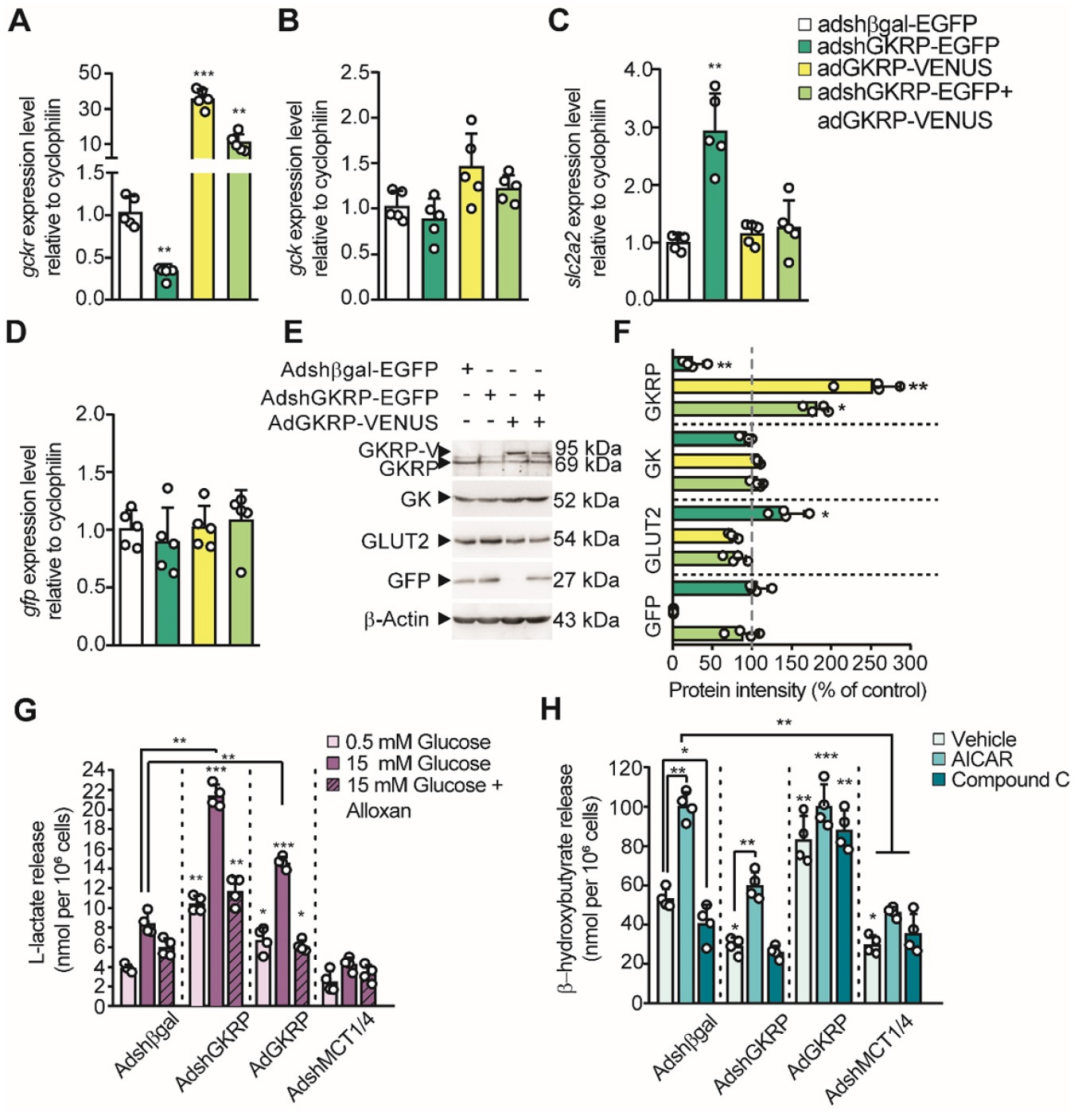
** Role of GKRP on tanycyte monocarboxylates production**. **A-D**: qPCR to evaluate the expression of *Gckr* (**A**), *Gck* (**B**), *Slc2a2* (**C**) and *Gfp* (**D**) in tanycyte cultures transduced with each virus (see legend). n = 5. **E**: Western blot to evaluate the protein expression of GKRP, GK, GLUT2, and GFP in transduced tanycytes. We used β-actin as the loading control. n = 4. **F**: Quantification of each band intensity with respect to loading control and as a percentage of the control condition (Adshβgal-EGFP transduction). **G-H**. Lactate (**G**) and βHB at 0.5 mM glucose(**H**) production by tanycytes following modification of both GKRP and MCT expression. * p < 0.05, ** p < 0.01, *** p < 0.001.

Considering that lactate and βHB production by tanycytes is regulated by glucose concentration in an opposing manner, we next proposed to evaluate the participation of GKRP in the production of these monocarboxylates, given its role as an inhibitor of GK. For this purpose, we produced four adenoviral vectors: AdGKRP-Venus and AdVenus-GKRP (exchanging the order of fusion proteins) for overexpressing GKRP, AdshGKRP-EGFP (AdGKRP-KD) for silencing GKRP, and Adshβgal-EGFP (Adβgal-KD) as a control. The INS-1 cell line, which does not express GKRP, was used to evaluate the expression of the Venus-fused GKRP vectors by immunohistochemistry. Both proteins showed a high degree of colocalization in INS-1 cells (Pearson's R of 0.97 and 0.94, respectively, Figure S2A-J). In addition, GKRP overexpression was confirmed using qPCR and immunoblotting (Figure S2J-K). Of these two forms of GKRP, only AdGKRP-Venus transduction significantly reduced the glucose phosphorylation at 10mM glucose (Figure S2L); therefore, we chose this construct for our subsequent studies (hereinafter termed AdGKRP). Efficient transduction of GKRP was maximal at 72 h (Figure S3B, E, H, J, K), with high cellular viability (Figure S3J-K); therefore, we used this time point to carry out all quantifications. Next, we tested the ability of the constructs to modify the levels of GKRP mRNA (*Gckr*) in tanycytes. We found a 3-fold reduction in the levels of *Gckr* after AdGKRP-KD transduction, while we observed a nearly 35-fold increase in *Gckr* in cells transduced with AdGKRP. Transduction with both viruses increased *Gckr* expression only by ~10 times with respect to control (Figure 4A), further demonstrating the ability of AdGKRP-KD to reduce *Gckr* levels. To evaluate if GKRP expression level affects glucosensing proteins in tanycytes, we quantified *Gck* (GK mRNA) and* Slcl2a* (GLUT2 mRNA) expression. After AdGKRP transduction, there was a trend towards an increase in *Gck* levels, albeit this effect was not significant compared to control s(Figure 4B,* p*: 0.052). However, *Slcl2a* expression showed ~3-fold increase after AdGKRP-KD transduction, and was not affected by *Gckr* overexpression or by double transduction of the constructs (Figure 4C). In contrast, GFP mRNA was similar across the different treatments (Figure 4D). Western blot analysis indicated that GKRP was reduced by 78% under AdGKRP-KD transduction, whereas it was increased by about 2.5-fold with AdGKRP (Figure 4E-F). In contrast, GK levels were not significantly changed under any treatment, while GLUT2 was increased ~45% with respect to control in GKRP-KD tanycyte cultures (Figure 4F). Since we obtained an efficient gain and loss of GKRP expression with these viruses, we next evaluated if GKRP affects monocarboxylate production by tanycytes.

In the presence of 15 mM glucose, tanycytes transduced with the control construct, Adβgal-KD, exhibited a similar increase in lactate release as controls (i.e. without transduction), when compared to the effect of 0.5 mM glucose. This 3-fold increase in lactate release was significantly reduced by alloxan, a GK inhibitor (Figure 4G). Furthermore, in tanycyte cultures transduced with AdGKRP-KD, alloxan also suppressed lactate release in response to 15mM glucose; however, there was a significant increase in lactate production in all conditions compared to controls, suggesting higher GK activity in the presence of AdGKRP-KD (Figure 4G). Unexpectedly, GKRP overexpression also resulted in an increase in lactate production, which was inhibited in the presence of alloxan (Figure 4G). These unexpected results could be partially explained by GKRP-dependent GK stabilization as previously proposed 20. As expected, transduction of tanycytes with a 1:1 mixture of AdMCT1-KD and AdMCT4-KD (to reduce expression of each MCT) abolished lactate release under all conditions evaluated, which is consistent with the notion that lactate export from tanycytes occurs through MCTs 14, 16.

Because tanycytes produce βHB in low glucose (Figure 1E), we evaluated whether GKRP affects the AMPK-dependent βHB production using AICAR and CpC at 5 mM glucose. Non-transduced cells and cells transduced with control adenovirus had the same response to these pharmacological agents (data no shown), whereas GKRP silencing evoked a decrease in βHB production compared to control transduced with AdβGal-KD (Figure 4H). Also, AICAR treatment decreased βHB release in tanycyte culture transduced with Ad-GKRP-KD, suggesting that GKRP is involved in the AMPK-dependent βHB production. However, GKRP overexpression resulted in an increase of βHB output even in the presence of CpC, suggesting that GKRP also could induce βHB production in an AMPK-independent mechanism. Like lactate, βHB release from tanycytes was significantly inhibited compared to AdβGal-KD, when both MCT1 and MCT4 were silenced (Figure 4H).

### *In vivo* modulation of GKRP expression alters the glucose response of orexigenic and anorexigenic neuropeptides

**Fig 5:**
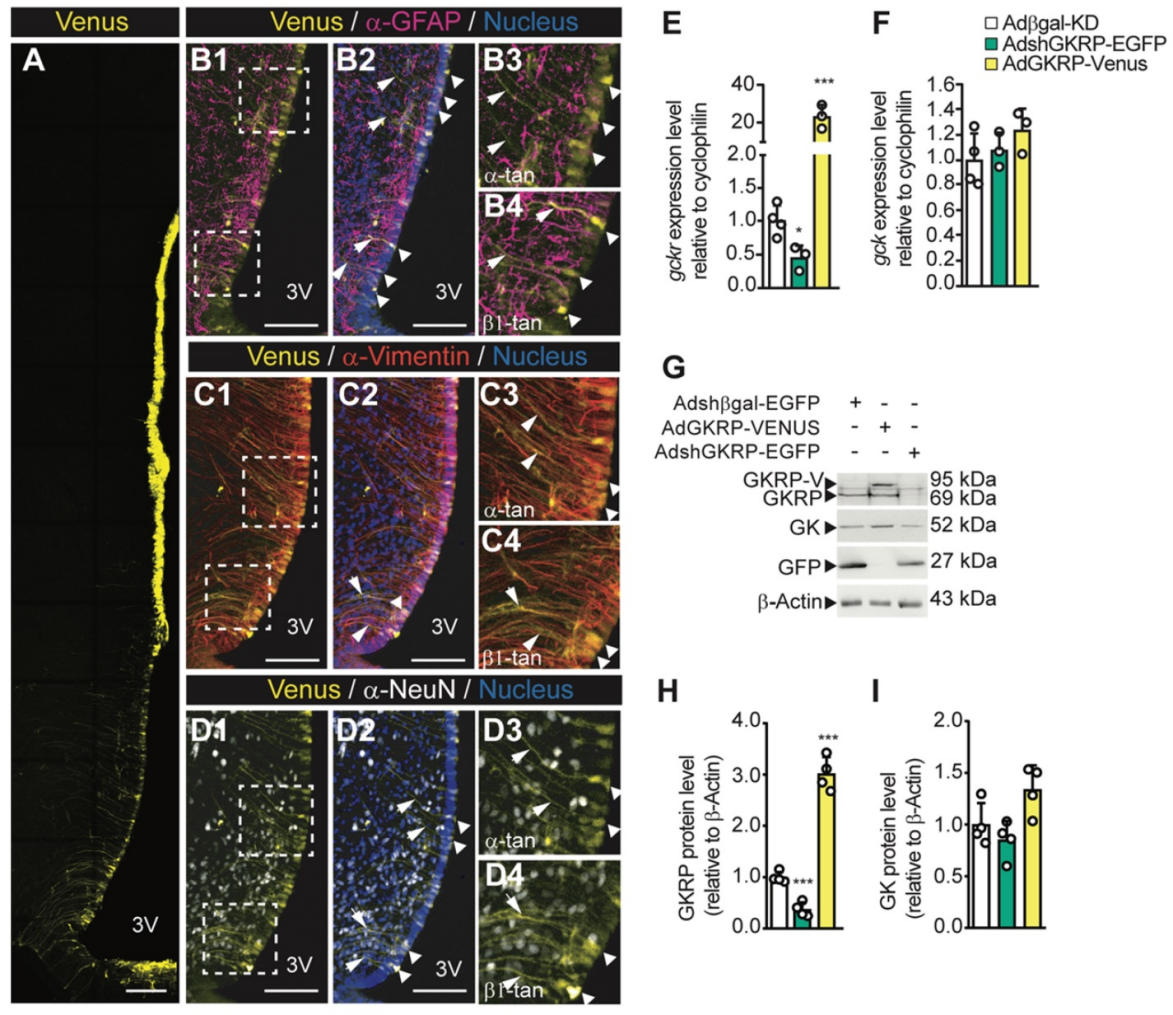
**Adenoviral transduction only affects the ventricular wall generating the expected effect on the basal hypothalamus**. Adult rats were stereotaxically cannulated and injected with 20 μL of each virus. 72 h post injection they were processed for immunohistochemistry (**A1-C4**, n = 3), RT-qPCR (**D, E**, n = 4), or Western blot (**F**, n = 4) analyses. Arrows indicate tanycyte processes positive for vimentin and the adenoviral reporter. Bar: 150 μm. **G-H**: Densitometric quantification of the band intensity for GKRP (**G**) or GK (**H**) with respect to β-actin and with respect to the control condition (Adshβgal-EGFP). * p < 0.05, *** p < 0.001.

To assess the physiological role of hypothalamic GKRP inhibition, we first examined the efficiency of transduction and the type of cells infected when we injected viral particles into the basal 3V. In AdGKRP-injected rats, confocal imaging revealed Venus fluorescence in the ventricular wall, including ependymocytes and all subpopulations of tanycytes (Figure 5A). The overlapping of Venus with GFAP, a glial marker, was restricted to ventricular cells and their processes but not in the subependymal band where astrocytes are localized, indicating that only α -and β-tanycytes were transduced by the construct (Figure 5B1-B4). Additionally, when we used anti-vimentin (expressed by tanycytes), we found a wide immunoreaction in the proximal part and processes of both α- and β-tanycytes (Figure 5C1-C2), which strongly overlapped with Venus labelling (Figure 5C3-C4). In contrast, we observed no overlap of Venus with the neuronal marker, NeuN, in basal hypothalamic neurons (Figure 5D1-D4). Similar results were obtained when we injected AdβGal-KD and AdGKRP-KD (data not shown), which agrees with our previous findings that show selective transduction in tanycytes using the same adenovirus backbone 11-14. Therefore, rats transduced with AdshGKRP and AdGKRP are referred to as GKRP^t^-KD and GKRP^t^-OE, respectively, for denoting tanycyte selective tropism. In order to evaluate GKRP and GK expression levels after the *in vivo* transduction, we first quantified the mRNA expression in periventricular hypothalamic samples. In GKRP^t^-KD rats, GKRP was significantly reduced by ~60% and ~80% at the mRNA (Figure 5E) and protein (Figure 5G-H) levels, respectively, compared with the control group. On the other hand, in GKRP^t^-OE rats, GKRP mRNA was increased 22 times (Figure 5E), while the protein reached ~3-fold increase (Figure 5G-H). GK mRNA expression was not significantly changed in both conditions (Figure 5F), but there was a slight trend toward an increased protein level upon GKRP overexpression (Figure 5I; 1.00±0.21 *vs.* 1.34±0.23, *p =* 0.071). Thus, these adenoviral vectors allowed us to generate highly selective transduction of tanycytes, and both GKRP and GK levels displayed similar changes to those detected *in vitro* after transduction with these vectors.

**Fig 6:**
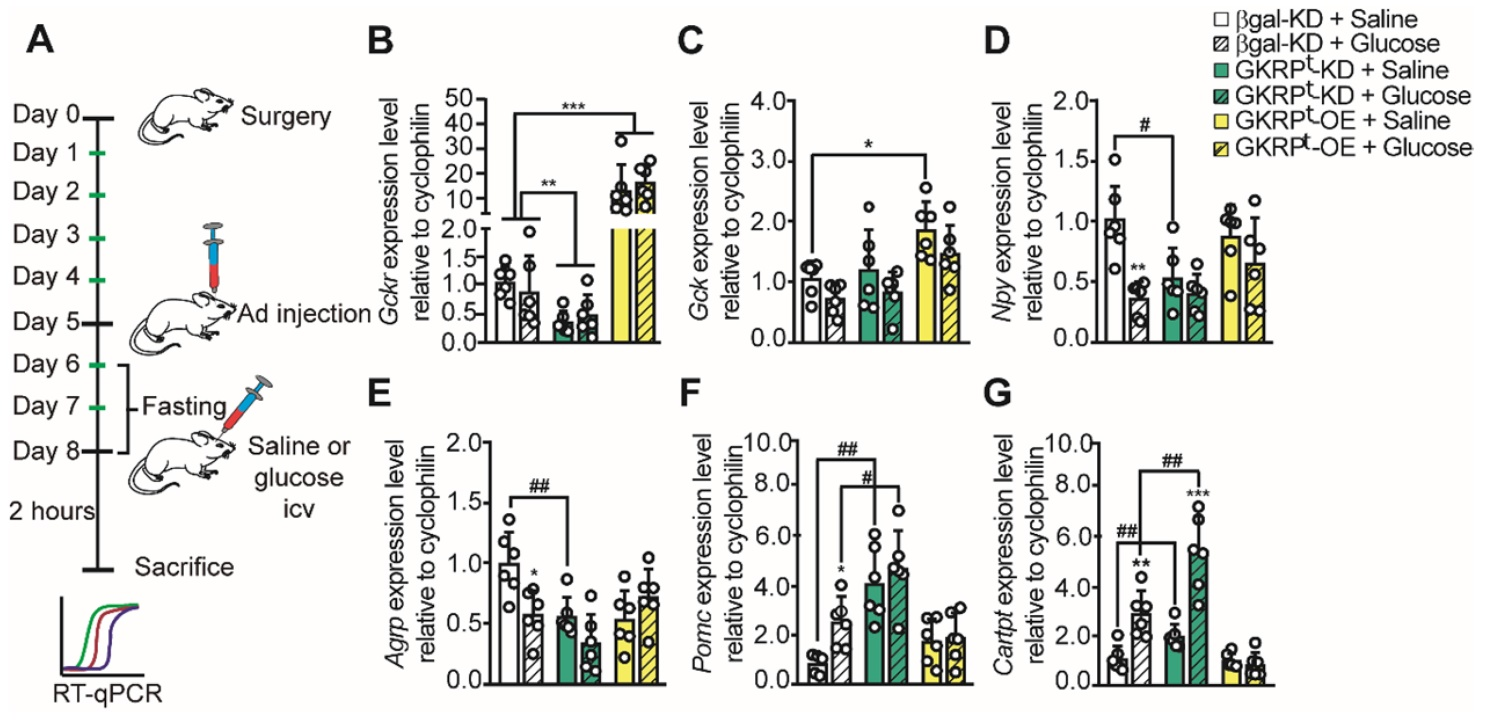
**Neuropeptide expression profile in response to glucose in rats with loss and gain of GKRP function**. According to the protocol in A, the expression of GKRP (B), GK (C), NPY (**D**), AgRP (**E**), POMC (**F**), and CART (**G**) mRNA was evaluated in fasted rats after 72 h transduction and 2 h of saline or glucose icv injection. n = 6 per condition. * p < 0.05, ** p < 0.01, *** p < 0.001, saline injection versus glucose. # represents an equivalent value of p but comparing saline or glucose with respect to the same condition in the control group.

Previous studies have shown that glucose injection into the 3V produced a significant decrease in orexigenic neuropeptides and an increase in anorexigenic neuropeptides in the ARC of fasted rats 11, 14, 18. Therefore, we tested if changes in tanycytic GKRP expression altered the normal response to glucose following the protocol shown in Figure 6A. Briefly, rats were allowed to recover for 5 days post-surgery, then they were icv-injected with each virus and treatments were performed 3 days post-transduction. To deplete the energy reservoirs, transduced rats were fasted for 48 h before stimulus injections. To minimize the number of animals for these experiments, we used AdβGal-KD as a unique control since the adenovirus that induced Venus expression showed the same neuropeptide expression profile in response to glucose (Figure S5). We first evaluated if glucose injection altered the amount of GK or GKRP mRNA in the hypothalamus. As shown in Figure 6B, *Gckr* was not affected by glucose injection. *Gck* did not change in response to glucose injection in GKRP^t^-OE rats but displayed a significant increase upon saline injection as compared to the control group (Figure 6C).

As expected, when we measured neuropeptide expression 2 h after icv injection of glucose in control rats, we detected a decrease in the mRNA for orexigenic neuropeptides and an increase in the mRNA for anorexigenic neuropeptides (Figure 6D-G). Remarkably, when GKRP was reduced by viral injection, Npy mRNA remained low even after 48 h-fasting (Figure 6D**,** green bar). On the other hand, *Npy* levels did not decrease significantly in response to glucose vs. saline injection in GKRP^t^-OE rats (Figure 6D). Similar results were obtained when we quantified *Agrp* (Figure 6E), suggesting that GKRP in tanycytes is necessary to increase hunger-inducing neuropeptides in fasting.

Analysis of anorexigenic neuropeptides revealed differences in their response to GKRP regulation. For example, *Pomc* did not increase in response to glucose in both GKRP^t^-KD and GKRP^t^-OE rats while the increase in *Cartpt* in response to glucose was abolished only in GKRP^t^-OE rats (Figure 6F-G). Interestingly, in GKRP^t^-KD rats, the amount of both anorexigenic neuropeptides was greater than in the control group (Figure 6F-G, #). In summary, GKRP^t^-KD leads to a profile of satiety-inducing neuropeptide expression, decreasing orexigenic peptides and increasing anorexigenic neuropeptides in response to glucose. In contrast, GKRP^t^-OE animals exhibited a more complex phenotype, characterized by an absence of glucose response for all measured neuropeptides.

### Modulation of GKRP expression changes feeding behavior in rats

**Fig 7:**
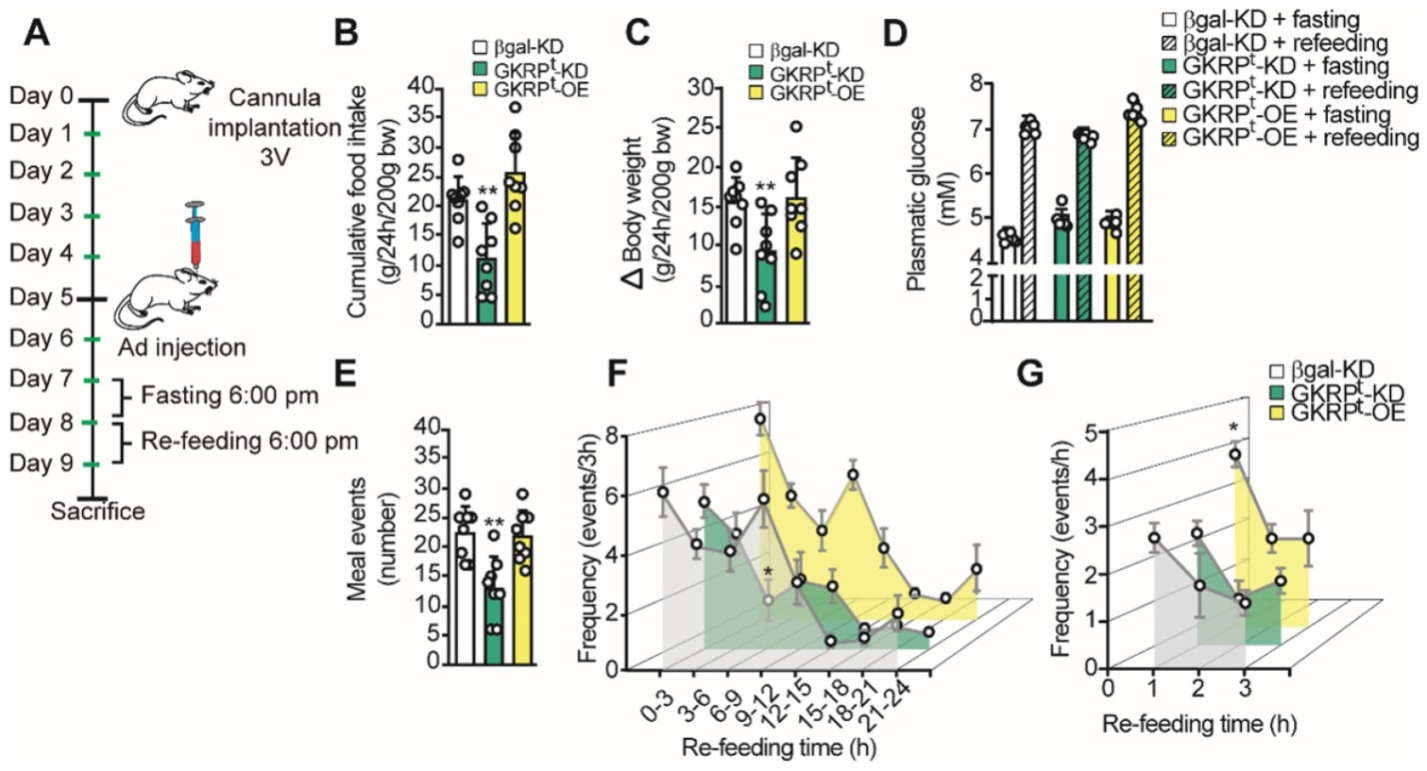
**Role of tanycyte GKRP on the macrostructure of feeding in rats. A**. Protocol for handling experimental animals. The rats were stereotaxically cannulated at baseline 3V to inject each virus after 5 days of recovery. After 2 days, the rats were subjected to a 24 h fast, a period after which they were offered 40 g of food for an additional 24 h. The animals were weighed before and after the re-feeding period. **B**. Cumulative food intake (CFI) of each animal for every 200 g of initial weight.** C**. Changes in body weight during the refeeding period normalized for every 200 g of initial body weight. **D**. Glycemia of animals before (filled bars) and after (hatched bars) refeeding in control (white bars), GKRP^t^-KD (green bars) and GKRP^t^-OE (yellow bars) animals. **E**. Number of feeding events in each group of animals. **F**. Feeding frequency of each group of animals determined as the number of feeding events for every 3 h of feedback. **G**. Frequency of feeding per hour during the first 3 h of refeeding. n = 8 per condition. * p < 0.05, ** p < 0.01.

To evaluate the role of GKRP on feeding behavior, we first measured food intake and body weight in GKRP^t^-KD and GKRP^t^-OE. For this, we followed the protocol scheme shown in Figure 7A where rats were fasted for 24 h before refeeding for an additional 24 h. GKRP^t^-KD rats displayed a significantly lower mean CFI (22.4 ± 3.0 vs. 11.0 ± 5.2g, *p =* 0.002; Figure 7B, green bars) and lower body weight gain with respect to the control group (15.4 ± 3.1 vs. 9.3 ± 4.7g, *p =* 0.008) (Figure 7C). In contrast, GKRP^t^-OE rats did not show significant differences in CFI or body weight compared to controls. It should be noted that rats transduced with each of adenovirus did not show differences in glycemia, either post fasting or in the refeeding period (Figure 7D). Total meal events, defined as the number of times that a feeding event occurs over 24 h, were significantly lower in GKRP^t^-KD rats compared with the control group (Figure 7E). In GKRP^t^-OE rats, the feeding events were not different from control. A detailed analysis over time (every 3 h) indicated that, unlike control rats, GKRP^t^-KD animals, but not GKRP^t^-OE, maintained a low frequency of feeding events between 6-9 h of the dark phase (Figure 7F). Because GKRP^t^-OE animals showed a slight tendency to increase the feeding frequency at the beginning of the refeeding period, we analyzed the frequency every 1 h and found a significant increase in this parameter only in the first hour of monitoring (Figure 7G).

**Fig 8:**
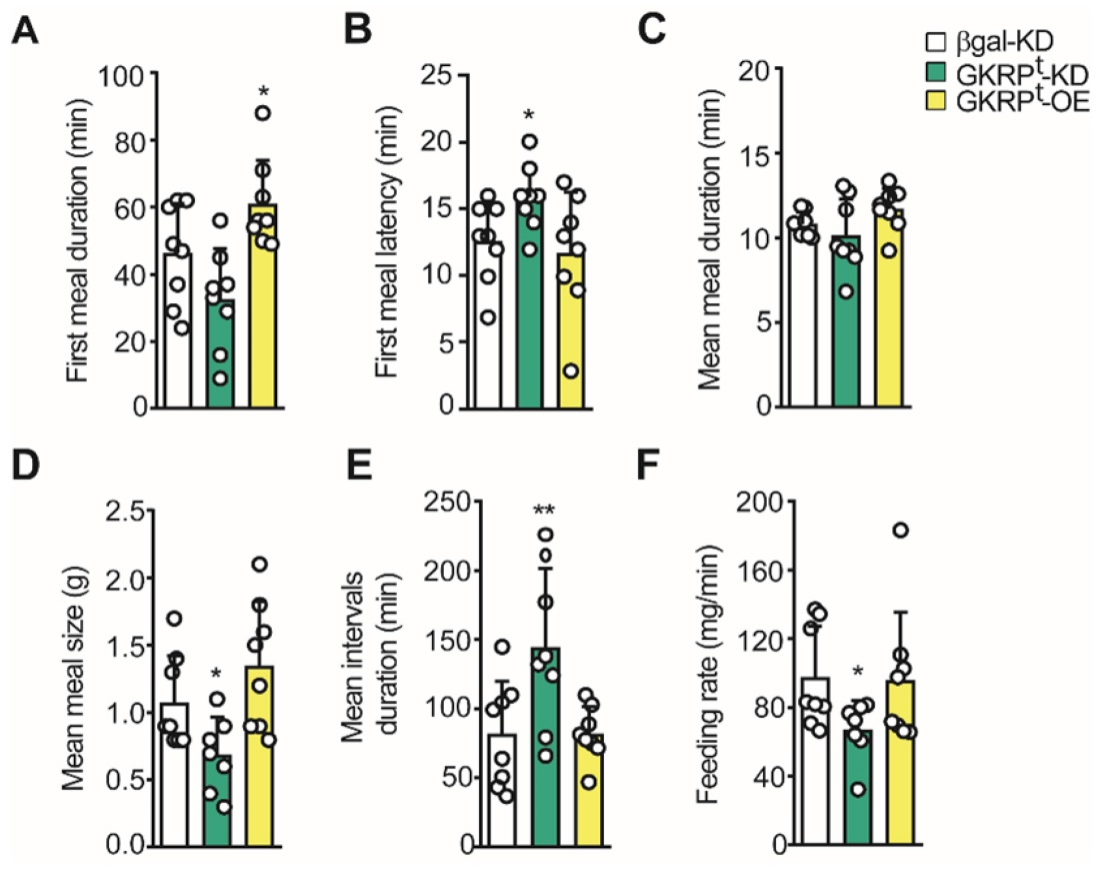
**Microstructure of feeding in GKRP KD and overexpressing rats**. **A**. Average duration of the first feeding event.** B**. Delay time (latency) at the beginning of the feeding after presenting the food. **C**. Mean duration of each feeding event.** D**. Mean meal size calculated as cumulative intake/number of feeding events. **E**. Mean duration of inter-meal intervals. **F**. Mean feeding rate in each experimental group. n = 8 per condition. * p < 0.05, ** p < 0.01.

Using the 24 h feeding data, we evaluated the microstructure of feeding. In the first term, we detected a significant increase in the first meal duration in GKRP^t^-OE rats, whereas GKRP^t^-KD rats did not show a difference in this parameter compared to the control (Figure 8A). This result is in line with the higher frequency of meals detected in the first hour of refeeding in GKRP^t^-OE rats (Figure 7G). However, in these animals, the latency of the first meal was not different from the control group, whereas GKRP^t^-KD rats take more time to start feeding (Figure 8B). The mean meal duration in 24 h of refeeding was not altered in any of the conditions (Figure 8C). Instead, when the mean meal size was evaluated, we obtained a reduction in GKRP^t^-KD rats compared to the control, without detectable changes in GKRP^t^-OE rats (Figure 8D). Because our previous results suggest an increase in satiety in GKRP^t^-KD rats (Figure 6-7), we analyzed the mean inter-meal interval duration in the whole monitoring period, since this parameter reflects satiety behavior. Indeed, the duration of the intervals between meals revealed a significant increase in GKRP^t^-KD rats with respect to the control group (Figure 8E, green bars), supporting higher satiety. GKRP^t^-OE rats did not display changes in mean inter-meal interval duration with respect to the control group (Figure 8E, yellow columns). Finally, the eating rate, which is a ratio between total food intake (g) and meal duration (min), was lower in GKRP^t^-KD rats, while GKRP^t^-OE rats displayed no difference (Figure 8F). In summary, the low eating rate, in addition to a high interval duration, is consistent with the decrease in food intake and lesser body weight gain in GKRP^t^-KD rats.

### Changes in feeding behavior of GKRP knockdown rats can be reverted by MCT1 silencing

**Fig 9:**
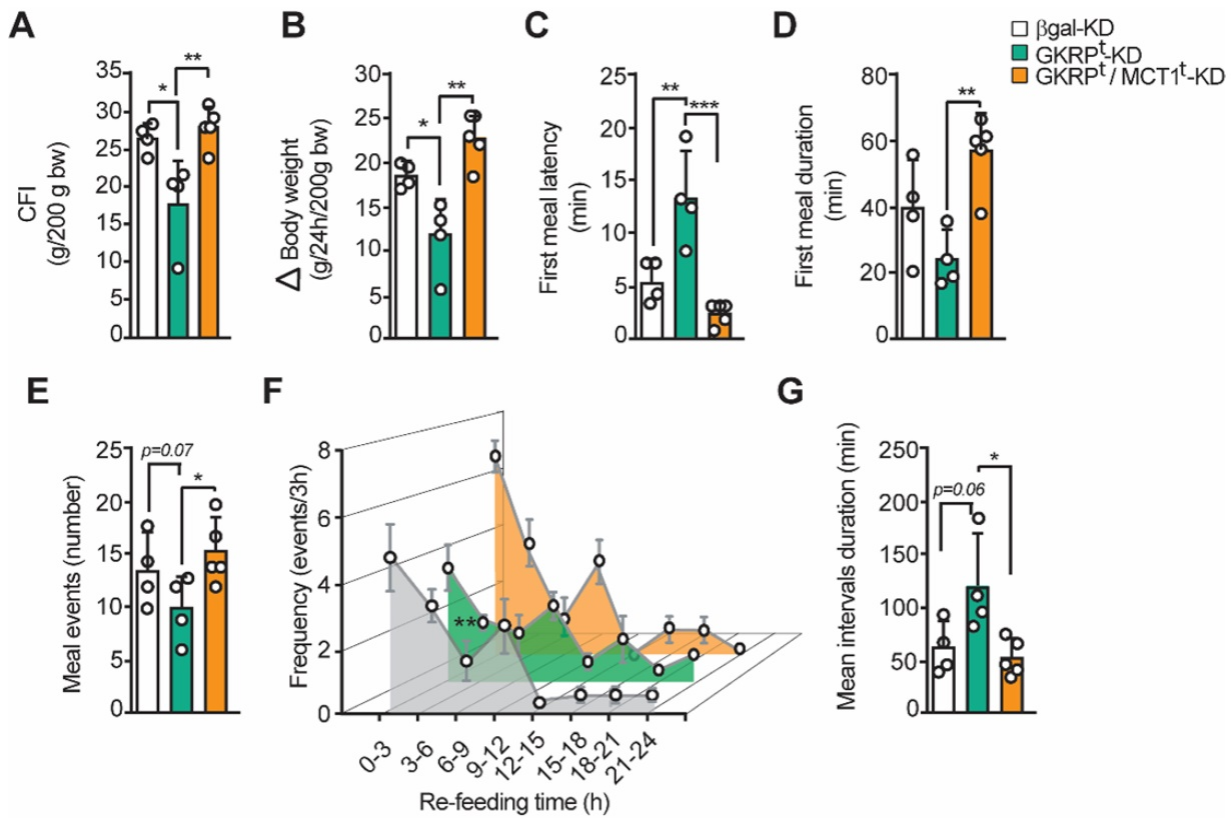
** The inhibition of MCT1 reverted the effect of GKRP knockdown over feeding behavior in rats. A**. Cumulative food intake (CFI) of each animal for every 200 g of initial weight.** B**. Changes in body weight during the refeeding period normalized for every 200 g of initial body weight. **C**. Delay time (latency) at the beginning of the feeding after presenting the food** D**. Average duration of the first feeding event **E.** Number of feeding events in each group of animals. **F**. Feeding frequency of each group of animals determined as the number of feeding events for every 3 h of refeeding. **G**. Mean duration of inter-meal intervals. n = 4 for GKRP^t^-KD and n = 5 for double knockdown. * p < 0.05, ** p < 0.01.

Our previous findings strongly suggest that the increase in satiety observed in GKRP^t^-KD rats is due to an increase in lactate release from tanycytes. To verify this hypothesis, we have developed a new feeding test but incorporating an experimental group in which we have jointly silenced GKRP and MCT1 by adenoviral injection. In line with our proposal, the food intake and body weight reduction observed in GKRP^t^-KD were completely reversed in the double knockdown group (Figure 9A-B). Furthermore, the lower avidity to eat by the GKRP^t^-KD animals after fasting was largely reversed in the double knockdown rats, as evidenced by a significant decrease in latency (Figure 9C) and a longer duration of the first meal (Figure 9D). These findings are aligned with a greater number of eating events in the double knockdown group versus the GKRP^t^-KD group (Figure 9E; 15.4 ± 2.4 *vs.* 10.0 ± 2.8, respectively). When analyzing the feeding frequency every 3 h in the double knockdown rats, no significant differences were observed in relation to the control group, while there was a significant decrease in this parameter in the GKRP-KD group in the first 9 h of refeeding (Figure 9F), as previously showed. Finally, the "satiated" phenotype of GKRP^t^-KD rats, as evidenced by significantly longer interval durations, was also completely reversed in double knockdown animals. Thereby, these results indicate that the anorexigenic response induced by GKRP silencing in tanycytes depends on the function of the MCT1 transporter, which in these circumstances mainly transports lactate.

## Discussion

Many studies have explored the hypothesis that lactate could play a role in inducing satiety 13, 21, and our data support this hypothesis. Consistent with this, our previous work in MCT knockdown-rats showed that tanycyte expression of MCT1 and MCT4 is required to maintain the response of POMC neurons to icv glucose and to normal food intake. Importantly, microstructure analyses demonstrated that this effect was due to a decrease in the induction of satiety 14. Similar results were obtained using electrophysiological recordings, showing that POMC neurons are activated by glucose and this response was lost when MCT1-4 were inhibited in tanycytes 19 or by direct incubation of hypothalamic slices with L-lactate 22. Here, we demonstrated that the modulation of glucose metabolism in tanycytes is important for the satiety response using behaviour studies.

We previously reported that tanycytes are high glycolytic cells where the inhibition of GK leads to increased feeding 12; therefore, we evaluated whether modulating the levels of GKRP, a GK regulator in tanycytes, affects eating behavior. Following tanycytic-GKRP modulation, we found significant changes in feeding behavior, which correlated with changes in hypothalamic neuropeptide expression. Importantly, our data strongly suggest that these changes depended on monocarboxylate production by tanycytes. Supporting this, tanycyte cultures produced more lactate when GKRP was silenced whereas βHB production increased following GKRP overexpression. Furthermore, we provide evidence for the first time that POMC neuron excitability is inversely regulated by lactate and βHB, supporting a new role for these monocarboxylates on neuronal coding during hunger or satiety.

**Fig 10:**
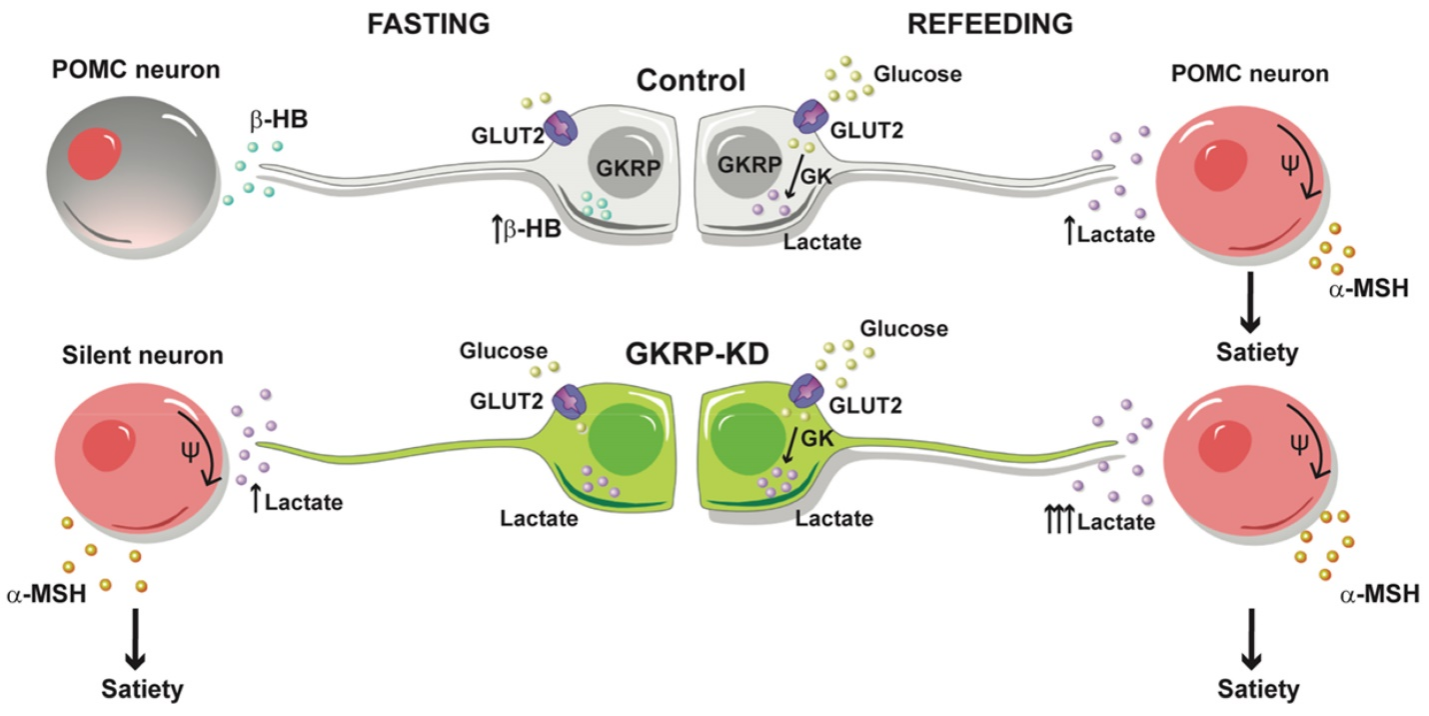
** Proposed tanycyte-POMC neuron communication under normal conditions and under GKRP silencing.** Under endogenous conditions, tanycytes sense hypoglycemia by producing βHB that reduces the activity of POMC neurons, contributing to the generation of hunger. After refeeding, ketogenesis stops and tanycytes incorporate glucose and generate satiety signals, such as lactate, which increases the excitability of POMC neurons. In contrast, the loss of GKRP function impacts a higher lactate production in fasting, especially after a meal, which exacerbates satiety.

Based on these results, we propose the model outlined in Figure 10 for the modulation of feeding behavior by tanycytes. In our model, glucose would be glycolytically metabolized after refeeding, thereby increasing lactate production in tanycytes, which upon release, favors the activation of nearby anorexigenic neurons that express POMC and CART neuropeptides. When we evaluated the neuropeptide expression profile induced by increasing brain glucose, a rise in the anorexigenic response in GKRP^t^-KD rats was detected. In agreement with this, GKRP^t^-KD animals ate significantly less and had significantly reduced body weight as compared to control rats, and they exhibited a behavior contrary to GK-KD rats 12. In a similar approach, Lam et. al. showed that the counterregulatory effect induced by a rise in central glucose was dependent on LDH-mediated hypothalamic lactate production 23. Moreover, icv injection of lactate evoked a significant decrease in food intake and body weight in rats 21, highlighting the role of glia-released lactate in the hypothalamic control of feeding.

Furthermore, we demonstrated for the first time that lactate and βHB modulate the excitability of POMC neurons in opposing ways. Accordingly, analysis of feeding microstructure in GKRP^t^-KD rats revealed an increase in the duration of the intermeal intervals, which was a powerful satiety indicator 24. Taken into account the high lactate production by GKRP-KD tanycytes and that this anorexigenic response was abolished by cotransduction with shMCT1 adenovirus, it is likely that satiety induction detected in GKRP^t^-KD rats is mediated by lactate. Surprisingly, GKRP^t^-KD rats also showed a satiety phenotype after fasting that could be attributed to the higher GK activity and increase in the expression of GLUT2 detected in tanycyte cultures. Considering that tanycytes have a privileged position as a mediator of ARC glucose sensitivity, and they are coupled with each other and other glial cells 25, this lactate-dependent satiogenic signaling could spread rapidly along the ARC. However, we cannot rule out the possibility that other metabolic or non-metabolic signals may be playing a role in these circumstances.

On the other hand, GKRP^t^-OE rats showed a loss in response to icv glucose and ate 30% more than controls at the onset of refeeding. Even though this behavior agrees with our previous results when we silenced GK in tanycytes 12, GKRP^t^-OE rats had a feeding behavior indistinguishable from control animals after the first hour of refeeding. In this context, it has been demonstrated that the GK-GKRP complex is dissociated in high glucose, increasing GK activity even in tissues with high GKRP expression 26-28. Therefore, in a high glycolytic cell, such as the tanycyte, lactate release after the first hour could compensate for the earlier intake observed in GKRP^t^-OE rats. In line with this possibility, we observed increased lactate production in GKRP-OE tanycyte cultures, which is consistent with our *in vitro* and *in vivo* observations of higher GK levels. Regarding the latter, Farrelly et al. described a positive posttranscriptional regulatory role for GKRP in maintaining GK levels and activity 29. Additionally, it has been proposed that GKRP anchoring to GK increases the half-life of this enzyme and enhances its stability 20. Thus, increasing glucose concentrations can destabilize the GK-GKRP complex, and nuclear-sheltered GK quickly returns to the cytoplasm to exert its catalytic action.

Fasting induces hepatic production of KB, including βHB, which plays a crucial role in supplying energy to the brain in this condition 30, 31. Here, we showed for the first time that tanycytes produce βHB in an AMPK-dependent manner, as previously reported in cortical astrocytes 15. Nevertheless, we showed that βHB-production from tanycytes was dependent on glucose concentration, but not from astrocytes, reinforcing the role of tanycytes as hypoglycemia sensor cells. Similarly, we observed an increase in βHB release in GKRP-overexpressing cultures, which is likely due to GK inhibition and the subsequent increase in the AMP/ATP ratio (Figure 8). Supporting these results, a recent report showed AMPK-dependent lipophagy in tanycytes 32, which could contribute to increasing FFA availability to initiate ketogenesis in fasting conditions. Since βHB was not significantly affected by CpC, we cannot rule out that GKRP could also play a role in an AMPK-independent manner.

Interestingly, the present electrophysiological results show that βHB inhibits POMC neurons, and its icv injection generates a short-term increase in dietary intake. Similarly, Carneiro et al. showed that intracarotid infusion of βHB evoked a long-term increase in food intake (up to 12 h), and it was associated with an increase in orexigenic neuropeptides 33. However, no effects were observed on the electrical activity of NPY neurons. Conversely, we have demonstrated a short duration and reversible orexigenic response by a single βHB-injection. This discrepancy may be due to a rapid turnover of CSF at the ventricular level, which undergoes a complete turnover 4-5 times per day 34. In addition, our *in vitro* data showed that βHB increases the expression of orexigenic neuropeptides, similar to results previously described *in vivo* 33, and it also inhibited POMC neurons, which could be responsible for hunger induction. Although the mechanism of POMC neuron inhibition was not addressed, it is widely known that the activation of NPY/AgRP neurons promotes POMC inhibition through GABA release 35, 36. Additionally, it should be noted that βHB is the endogenous ligand of HCA2R, a G_i/o_-coupled receptor whose expression has been detected in the hypothalamus of rodents 37, and its activation reduces Ghrh release 38. Therefore, it is plausible to argue that POMC neurons can be directly or indirectly inhibited through βHB extracellular signaling after fasting periods. However, the short-term orexigenic effects we detected were rapidly reverted. Interestingly, POMC neurons express MCT2 39, so it is possible that βHB could be incorporated in these neurons and exert a lactate-like role, increasing melanocortin release via ATP production. This possibility is in line with our results in primary cultures of neurons, in which low, but not high, concentrations of βHB modified *Pomc* expression. Intriguingly, in our electrophysiological recordings, the inhibition of POMC neurons by βHB reversed to a depolarization during its washout. The mechanism for this depolarization is not clear, but it could be attributed to βHB uptake and metabolism to ATP. Nevertheless, independent of the mechanism of POMC inhibition, a very similar feeding response was detected in both GKRP^t^-OE and icv-injected rats with βHB, i.e. orexigenic at short-term and anorexigenic at longer. Considering that tanycytes release βHB when GKRP is overexpressed, a large part of these effects may be due to the tanycytic production of βHB.

Interestingly, hypothalamic KB production induced by high-fat diets promotes a lower body weight gain in rats after 3 h of food consumption 40, which is in-line with our long-term results when we injected βHB into the ventricle. This anorexigenic effect of βHB may shed light on why a ketogenic diet or intermittent fasting are effective strategies to reduce body weight 41-44. In addition, numerous studies have demonstrated that KB have other beneficial effects on brain health, particularly by improving hypothalamic energy homeostasis 40, preventing neuroinflammation 45-47, and protecting neurons from death 48. Our results highlight the notion that KB are more than just cellular fuels and can exert profound effects on energy homeostasis. Further studies will help us to elucidate more finely the temporal profile of this tanycyte-dependent regulation.

Altogether, our results indicate that the inhibition of GKRP function in tanycytes could serve as a therapeutic target to boost satiety in patients with obesity problems. In this context, highly effective drugs have been developed that dissociate the GK/GKRP complex in the liver, thereby stimulating glucose phosphorylation and reducing blood glucose levels in diabetic animals 5, 49. Our present work provides evidence that the inhibition of GKRP, specifically in tanycytes, reduces food intake by increasing satiety. Additional studies will help elucidate how the regulation exerted by GKRP is orchestrated with glial production of KB in the context of anti-obesity therapy. These results reinforce the leading role played by tanycytes in the regulation of neurons responsible for regulating hunger and satiety and may open the door to new experimental strategies targeting the GKRP protein.

## Materials and methods

### Ethics statement

All animals were handled in strict accordance with Animal Welfare Assurance (permit number 2010101 A), and all animal work was approved by the appropriate Ethics and Animal Care and Use Committee of the Universidad de Concepción, Chile, and the University of Maryland, College Park MD, USA. One hundred and five male adult Sprague-Dawley rats of 250-300 g and fifty POMC-EGFP mice (C57BL/6J-Tg(Pomc-EGFP)1Low/J) were used in all experiments. Animals were housed in a separate animal room with constant temperature (21 ± 2 °C) and a controlled 12-h light/12-h dark cycle. Animals had free access to a standard rodent diet (Lab Diet, 5P00 Prolab RMH 3000, Purina Mills, St. Louis, MO) and tap water, except in those experiments in which fasting was induced.

### Preparation of adenovirus particles

Serotype 5 ΔE1, E3-based replication-deficient adenoviruses were generated as previously described 13. To produce AdshGKRP-EGFP, oligonucleotides targeting rat GKRP were designed and selected using the Genebank accession number KJ026952.1, sense shRNA-GKRP 5′-CGC GCC GCC AAA GCA GAT GCA GAG AAA T-3′ and antisense shRNA-GKRP 5′-TTA AAA AAA CAA AGC AGA TGC AGA GAA A-3′ sequences that shared no homology with other rat coding sequences by BLAST analysis. A ring sequence of nine base pairs (TTC AAG AGA) existed between the sense and antisense strands. Control shRNA oligonucleotides were designed and selected to target β-galactosidase from *E. coli*: sense 5′-CGC GCC AAG GCC AGA CGC GAA TTA TTT CAA GAG AAT AAT TCG CGT CTG GCC TTT TTT TTT TAA T-3′ and antisense 5′-TAA AAA AAA AAG GCC AGA CGC GAA TTA TTC TCT TGA AAT AAT TCG CGT CTG GCC TTG G-3′. The expression cassette was then cloned into the adenoviral shuttle vector. All shRNAs (Invitrogen, Rockville, MD, USA) were synthesized and designed to contain both AscI and PacI restriction enzyme sites (New England Biolabs, Ipswich, MA, USA), which were used for ligation into pDC311.2-OFF-EGFP downstream of the human H1 promoter. To produce adenoviruses capable of overexpressing GKRP, we produced a GKRP-Venus cassette by incorporating both BamHI and KpnI at the 5'-and-3' end of tanycyte GKRP cDNA to clone it into the adenoviral shuttle vector. The plasmid was then co-transfected with the Ad genomic plasmid, pBHGlox∆E1,3Cre (Admax system, Microbix Biosystems, Ontario, Canada) into HEK293A cells. Virus particles were released by heat shock, and cell debris was removed by centrifugation for 5 min at 5000× g. The particles were recovered from the supernatant by filtration through a 0.45-µm filter. The resulting adenoviral expression vectors (AdshGKRP-EGFP, Adshβgal, and AdGKRP-Venus) were titered by EGFP or Venus expression using the Adeno-XTM Rapid Titer Kit Protocol (Clontech, Mountain View, CA, USA).

### Cannula implantation

Rats were handled every day for one week before the experiments to get acclimated to the researchers and the experimental procedures. Rats were anesthetized with an intraperitoneal injection of ketamine (90 mg/kg) and xylazine (10 mg/kg). The fur at the top of the head was removed to expose the area to be incised. A hole was drilled in the skull, and a guide cannula (28-gauge stainless steel; Plastics One, Roanoke, VA) was stereotaxically implanted in the 3V of the rat (anterior-posterior from bregma -3.14 mm, medial-lateral from midsagittal sinus 0.0, and dorsal-ventral from the top of the skull 9.2 mm). The guide cannula was secured to the skull using 3/32 mm mounting screws and dental acrylic. A removable dummy cannula (28-gauge stainless steel; Plastics One) was fit into the cannula guide and sealed the opening of the guide cannula throughout the experiments except when it was removed for the injections. After the surgery, rats were single-housed and allowed to recover for 5 days before adenovirus injection.

### Icv injections of adenoviruses and immunohistochemistry

Rats were anesthetized with isoflurane and then injected into the 3V with either 25 µL of the control Ad-shβGal-EGFP stock solution (10^9^ IFU/mL) or 25 µL of the Ad-shGKRP-EGFP stock solution (2 × 10^8^ IFU/mL) for the experimental group at 2.5 µL/min. We were not able to further concentrate the adenovirus in order to inject it in a lower volume. Brains were collected for immunohistochemistry at 18, 24, and 48 h. The rat brains were fixed in 4% paraformaldehyde (PFA) by immersion for 48 h. After fixation, thick frontal sections of the hypothalamus (40 µm) were cut with a cryostat and subsequently processed free-floating. Tissues were immunoassayed with mouse anti-vimentin (1:200, DAKO, Carpinteria, CA, USA), rabbit anti-glial fibrillary acidic protein (GFAP; 1:200, DAKO) and rabbit anti-NeuN (1:1000, Abcam, Cambridge, MA, USA) antibodies diluted in Tris-HCl buffer (pH 7.8) containing 8.4mM Na_2_HPO_4_, 3.5mM KH_2_PO_4_, 120mM NaCl, and 1% bovine serum albumin (BSA). Sections were incubated with the primary antibodies overnight at room temperature in a humid chamber. After extensive washing, sections were incubated for 2 h at room temperature with Cy2-, Cy3- or Cy5-labeled secondary antibodies (1:200; Jackson ImmunoResearch, West Grove, PA, USA). These samples were counterstained with the DNA stain TOPRO-3 (1:1000; Invitrogen). The slides were analyzed using confocal laser microscopy (LSM 700, Zeiss, CMA-Biobio). Colocalization of different markers was assessed by measuring the Pearson's correlation coefficient Rr. An Rr value of '1' indicates complete colocalization, and an Rr value of '0' indicates no specific colocalization.

### Glycemia measurement

Through a sterile lancet puncture in the lateral vein of the rat tail, the plasma glucose concentration (mg/dL) was determined in each animal before and after each procedure to analyze the generation of hypoglycemia in fasting periods and to verify the correct state of health of the animals used in the studies. For this, the Accu-Chek Go glucometer (Roche) was used.

### Hexokinase assays

To evaluate the effect of GKRP overexpression on GK activity, 832/13 cells were transduced with 5 x 10^7^ IFU/mL each adenovirus and incubated for 72 h. After that, cells were lysed by ultrasound, and HK activity was promptly determined as previously described with slight modifications 50. Briefly, a glucose 6-phosphatase (G-6P) dehydrogenase-coupled reaction was used, and the activity was followed by measuring the increase in absorbance at 340 nm after 5 min incubation at 37°C. The reaction mixture consisted of 200mM Tris-HCl buffer (pH 7.5), 2mM MgCl_2_, 1mM DTT, 1mM ATP, 0.5mM NADPH, 1-30mM glucose, and 1 U/mL of G-6P dehydrogenase (Sigma-Aldrich). For specific activity determination, we used 0.5 mg/mL of total protein in the reaction mixture, and the Prism software was used for data analysis (GraphPad, Inc.). To determine the reaction velocity for each absorbance, we made a calibration curve with G-6P as a substrate.

### Electrophysiological recordings

Hypothalamus slices from POMC-EGFP mice (Jax Catalog # 009593) were prepared in cold oxygenated aCSF containing low Ca^2+^ concentration (0.5mM) and high Mg^2+^ concentration (6 mM). Sections of 250 µm were kept for 30 min at 35 ° C in normal aCSF (125mM NaCl, 26mM NaHCO_3_, 1.25mM NaH_2_PO_4_, 2.5mM KCl, 2mM CaCl_2_, 1mM MgCl_2_, 1mM myoinositol, 0.4mM ascorbic acid, 2mM sodium pyruvate and 0.5mM glucose) and were continuously oxygenated (95% O_2_ - 5% CO_2_), at a pH of 7.4 and an osmolarity of 305 mOs. POMC neurons were identified based on EGFP fluorescence, visualized using an Olympus BX51W1 microscope, and recorded using an EPC10 dual amplifier (HEKA) in whole-cell current-clamp mode. Electrical stimulation and recordings were performed using PatchMaster software. The experiments were performed at room temperature with a perfusion rate of 2-3 mL/min and using standard patch pipettes (4-8 MΩ), evaluating neuronal activity in response to 10 mM L-lactate or 2mM βHB. Data analysis was performed with Igor Pro 8 software.

### Primary culture of tanycytes

Highly enriched primary tanycyte cultures from 1-day postnatal brains (10-12 rats) were isolated and immunocharacterized following the method described previously 13, 16, 50, 51. Briefly, the hypothalamus was removed and further dissected to obtain a region close to the ependymal layer. The dissection was carried out with the samples submerged in dissection buffer containing 10mM HEPES (pH 7.4, 340 mOsm/L). Trypsinized tissue was transferred to planting medium containing MEM (Invitrogen) with 10% (v/v) fetal bovine serum (FBS) (Thermo Fisher Scientific Inc., Waltham, MA, USA) and 2 mg/mL DNAse I (Sigma-Aldrich). Cells were seeded at 1.2 x 10^5^ cells/cm^2^ in culture dishes treated with 0.2 mg/mL poly-L-lysine (Sigma-Aldrich). After 2 h, the culture medium was changed to MEM (5mM glucose) supplemented with 10% FBS, 2mM L-glutamine, 100 U/mL penicillin, 100 mg/mL streptomycin, and 2.5 mg/mL fungizone (Thermo Fisher Scientific, Inc). Cells were cultured in the same dish for 2 weeks and the medium was changed every 2 days. To study adenoviral transduction rate and viability, cells were grown in 2% FBS DMEM and then transduced with 5x10^7^ IFU/mL for 24, 72, and 96 h before immunocytochemistry analyses.

### Primary culture of astrocytes

Primary astrocyte cultures were prepared from rats 1 to 3 days postnatal, which were sacrificed by guillotine to obtain brains. Meninges-free cortexes were dissected and minced into small pieces in the presence of PBS. Then, these tissue pieces were subjected to enzymatic digestion for 15 min at 37 ° C in the presence of 0.25% w/v trypsin and 0.2% EDTA. Then, mechanical disintegration was performed in DMEM medium supplemented with 10% FBS, 2mM glutamine, 100 U/mL penicillin, 100 µg/mL streptomycin, 2mM glucose and 2.5 µg/mL fungizone. The cells were then seeded in culture plates at an approximate density of 1 x 10^5^ cells/mL. After 4 h post seeding, the culture medium was completely replaced. Subsequently, the culture medium was replaced every 48 h for 14 days.

### Primary culture of hypothalamic neurons

A hypothalamic neuron-enriched primary culture was prepared from E18 Sprague-Dawley rats and maintained in culture at 37 °C and 5% CO_2_. Briefly, the hypothalamic region of the brain was microdissected at 4 °C in a buffer containing 10mM HEPES (pH 5 7.4; 340 mOsm/L). Subsequently, the tissue was subjected to enzymatic desegregation for 10 min at 37 °C in 0.25% trypsin (Invitrogen) and 0.20% EDTA (Sigma-Aldrich). After the tissue was transferred to MEM (Invitrogen) with 10% (v/v) FBS (Thermo Fisher Scientific Inc., Waltham, MA, USA) and 2 mg/mL DNAse I (Sigma-Aldrich), mechanical disaggregation was performed. The cells (5 x 10^5^ cells/cm^2^) were plated on coverslips (18 mm in diameter) that were coated with 0.2 mg/mL poly-L-lysine (Sigma-Aldrich) in 12-well plates (Corning Costar, NY, USA). After 30 min, Neurobasal medium supplemented with B27, 2mM glutamine, 100 U/mL penicillin, 100 mg/mL streptomycin, and 2.5 mg/mL Fungizone (all Invitrogen) was added. After 48 h, the cultures were treated with 15 mg Ara-C (Sigma-Aldrich) and maintained in culture for 7 days until subsequent experiments.

### Monocarboxylate release determination

For L-lactate release determination, tanycyte primary cultures (10 days *in vitro*) were seeded at 2.5 x 10^6^ cells/cm^2^ for an additional 2 days at 5mM glucose and then incubated with different glucose concentrations in phenol red-free DMEM medium for 60 min. To evaluate the role of GKRP or MCTs, these cells were pre transduced with each adenoviral preparation (5 x 10^7^ IFU/mL) for 72 h. For βHB release determination, a similar approach was carried out in tanycyte and astrocyte cultures, but cells were incubated with different glucose concentrations or modulators of AMPK activity. At the end of this period (60 min), 500 µL of each culture medium was stored at -80 ° C until processing. Both lactate and βHB analyses were performed by colorimetric assays using an L-lactate assay kit (Cayman Chemical cat # 700510) or β-Hydroxybutyrate (Ketone Body) Colorimetric Assay Kit (Cayman Chemical cat # 700190), following the instructions of the manufacturer.

### Immunocytochemistry

Cultured cells were grown on poly-L-lysine-coated (Sigma-Aldrich) glass cover slides in 24-well plates, fixed with 4% PFA in PBS for 30 min, washed with Tris-HCl buffer (pH 7.8) and incubated in the same buffer containing 1% BSA and 0.2% Triton X-100 for 5 min at RT. Samples were then incubated with mouse anti-vimentin (1:200, Dako) to evaluate the purity of tanycyte cultures. Cells were next incubated with Cy2- or Cy3-labeled secondary antibodies (Jackson ImmunoResearch Laboratories), counterstained with the DNA stain, TOPRO-3 (1:1000, Invitrogen), and analyzed using confocal laser microscopy (Carl Zeiss, LSM700).

### Reverse Transcription- Quantitative Polymerase Chain Reaction (RT-qPCR)

For hypothalamic neuropeptide expression analysis, the tissues were processed for RT-qPCR analysis as previously described 12, 13. Briefly, 48 h after adenovirus injection, rats were fasted for 24 h. Rats were then anesthetized with isoflurane and icv injected with 10 μL of saline buffer (128mM NaCl, 3mM KCl, 1.3mM CaCl_2_, 1.0mM MgCl_2_, 1.3mM NaH_2_PO_4_, 21mM Na_2_HPO_4_, pH 7.4 and 320 mOsm) or 10 μL of 50mM D-glucose diluted in the same buffer (320 mOsm, pH 7.4) at a rate of 2.5 μL/min. By using a binocular loupe, periventricular hypothalamic samples were collected 2 h post-glucose or saline injection for the mRNA analysis expression. RT-qPCR analysis was used to measure the expression of hypothalamic cyclophilin (the housekeeping gene), GK, NPY, CART, POMC, and AgRP. After the experimental treatments, the brain of each rat (n = 6 per condition) was removed and the hypothalamic area was isolated and further dissected to obtain a region close to the 3V ependymal layer. All microdissections were performed closest to the basal 3V and under a stereomicroscope Leica M80 (Leica, Wetzlar, Germany). Total RNA from the hypothalamus was isolated using TRIzol (Invitrogen) and treated with DNase I (ThermoFisher Scientific) to remove genomic DNA contamination. A total of 2 µg of RNA from each sample was reverse transcribed into cDNA according to the manufacturer's protocol of M-MULV reverse transcriptase (ThermoFisher Scientific). Parallel reactions were performed in the absence of reverse transcriptase to control for the presence of genomic DNA. qRT-PCR reactions were prepared with a Brilliant II SYBR Green QPCR Master Mix kit (Agilent Technologies, Inc., Santa Clara, CA, USA) in a final volume of 12.5 µL containing 1x SYBR green Master Mix, 1 µL cDNA sample and 500nM of the following sets of primers: cyclophilin, sense 5′-ATA ATG GCA CTG GTG GCA AGT C-3′ and antisense 5′-ATT CCT GGA CCC AAA ACG CTC C-3′ (expected product of 239 bp); GK, sense 5′-AAA GAT GTT GCC CAC CTA CGT GCG-3′ and antisense 5′-ATC ATG CCG ACC TCA CAT TGG C-3′ (expected product of 510 bp); NPY, sense 5′-TGT TTG GGC ATT CTG GCT GAG G-3′ and antisense 5′-CTG GGG GCA TTT TCT GTG CTT TC-3′ (expected product of 203 bp); AgRP, sense 5′-GCA GAC CGA GCA GAA GAT GTT C-3′and antisense 5′-GTA GCA CGT CTT GAA GAA GC GG-3′ (expected product of 186 bp); POMC, sense 5′-CTC CTG CTT CAG ACC TCC ATA GAC-3′ and antisense 5′-AAG GGC TGT TCA TCT CCG TTG-3′ (expected product of 164 bp) and CART, sense 5′-TCT GGG AAG AAG AGG GAC TTT CGC-3′and antisense 5′-TCC ATT TGT GTT GCT TTG GGG TG-3′ (expected product of 137 bp). All reactions were performed with an initial denaturation of 5 min at 95 °C, followed by 40 cycles of 30 s at 95 °C, annealing for 30 s at 55 °C, and extension for 1 min at 72 °C in an Mx3000 P qPCR System (Agilent Technologies). Ct values of each mRNA obtained from three different experiments were normalized according to the 2-ΔΔCt method using cyclophilin as a reference gene.

### Immunoblotting

For protein analysis, hypothalamic samples were collected 48 h post-adenoviral injection. Small pieces of tissue or cultured primary cells were homogenized in RIPA buffer supplemented with complete protease and phosphatase inhibitor cocktail (ThermoFisher Scientific) and then sonicated three times on ice at 300 W (Sonics & Material INC, VCF1, Connecticut, USA) for 10 s. After isolation by centrifugation at 8,000× g for 10 min, the proteins were quantified and resolved by SDS-PAGE (50 µg/lane) in a 10% (w/v) polyacrylamide gel, transferred to PVDF membranes (0.45 µm pore, Amersham Pharmacia Biotech., Piscataway, NJ, USA), and probed with mouse anti-GK (1:2000, Santa Cruz Biotechnology, CA, USA), rabbit anti-GKRP (1:1000, Santa Cruz Biotechnology), rabbit anti-GLUT2 (1:1000, Alpha Diagnostics), mouse anti-EGFP (1:2000, Santa Cruz Biotechnology), rabbit anti-MCT1 (1:1000, Millipore), rabbit anti-total AMPK (1:1000, Cell signaling), rabbit anti-phospho-AMPK (1:1000, Cell signaling) and HRP-conjugated anti-β-actin (1:10000, Santa Cruz Biotechnology) antibodies. After extensive washing, the PVDF membranes were incubated for 2 h at 4 °C with peroxidase-labeled anti-rabbit IgG (1:10000; Jackson ImmunoResearch). The reaction was developed using an enhanced chemiluminescence (ECL) Western blotting analysis system (Amersham Biosciences). Negative controls consisted of incubating the membrane in the absence of primary antibodies. The images shown are representative of at least three analyses performed on samples from at least three separate experiments. β-actin expression levels were used as a loading control, and GFP expression levels were used as a transduction control.

### Food intake monitoring and feeding behavior analysis

At 24 h post-adenoviral injection, rats were subjected to a 24-h fasting period followed by a 24-h refeeding period for the feeding behavior analysis. Food intake was monitored during the refeeding period, at the beginning of dark phase, by providing rats with pre-weighed rat chow and weighing it again after 24 h (Figure 7A, schematic representation). Food intake was expressed as g consumed per 200 g of body weight. Every interaction with the feeder was recorded by a computerized data acquisition system (VitalView, Respironics, Inc., Murraysville, PA, USA), registering frequency and time of permanency in the feeder.

A meal was defined as a bout that was larger than 10 s into the feeder, and these meals were separated from other feeding bouts by more than 10 min of inter-meal interval. Except for the first meal, when the bout was longer than 30 min, two meals were considered. Meal patterns calculated included the following: duration of the first meal (min), frequency of meals (number, each 1/3 h), cumulative eating time as the total time spent in the feeder (min), duration of inter-meal intervals (min), and number of meal intervals. The inter-meal interval was calculated as the time between the end of one meal and the initiation of the next one. The mean meal size was determined as the total food intake (g) divided by frequency. The mean meal duration was calculated by dividing the total meal duration (in min) by meal frequency, and the eating rate was estimated by dividing total food intake (mg) by meal duration (min).

### Statistical analysis

For each data group, results were expressed as mean ± standard deviation (SD) of the mean, and n refers to the number of animals that were used. For statistical analysis, each treatment was compared with its respective control. Differences between two groups were assessed using the Student's t-test. Differences between more than two groups were assessed using one way-ANOVA. Another specific statistical tests or post-tests are described in each figure legend. Differences were considered significant when *p* < 0.05. The statistical analyses were performed using GraphPad Prism 8.01 Software (GraphPad Software Inc., San Diego, CA, USA).

## Supplementary Material

Supplementary figures.
