# Supplementary material for: GKRP-dependent modulation of feeding behavior by tanycyte-released monocarboxylates
Source: Theranostics. 2022 Jan 3;12(4):1518–36. doi: 10.7150/thno.66634 (PMC8825604; doi:10.7150/thno.66634)
Supplement: Supplementary file 1 — Supplementary figures. [file thnov12p1518s1.pdf]

GKRP expression inversely modifies L-lactate and β-hydroxybutyrate production in tanycytes with effects on feeding behavior.

Magdiel Salgado[1], Roberto Elizondo-Vega[1], Pablo S Villar[2], Macarena Konar[1,5], Scarlet Gallegos[5], Estefanía Tarifeño-Saldivia[1,3], Patricia Luz-Crawford[4], Luis G. Aguayo[5], Ricardo C. Araneda[2], Elena Uribe[3] and María Ángeles García-Robles[1*]

[1]Departamento de Biología Celular, Facultad de Ciencias Biológicas, Universidad de Concepción, Chile. [2]Department of Biology, University of Maryland, USA. [3]Departamento de Bioquímica y Biología Molecular, Facultad de Ciencias Biológicas, Universidad de Concepción, Concepción, Chile. [4]Centro de Investigación Biomédica, Facultad de Medicina, Universidad de Los Andes, Santiago, Chile

Keywords: Tanycyte, GKRP, lactate, β-hydroxybutyrate, feeding behavior, obesity

*Address correspondence and reprint requests to M.A. García-Robles, Laboratorio de Biología Celular, Departamento de Biología Celular, Facultad de Ciencias Biológicas, Universidad de Concepción, Concepción, Chile. mgarcia@udec.cl, Orcid 0000-0001-8482-0361, Phone number: 56-41-2203805

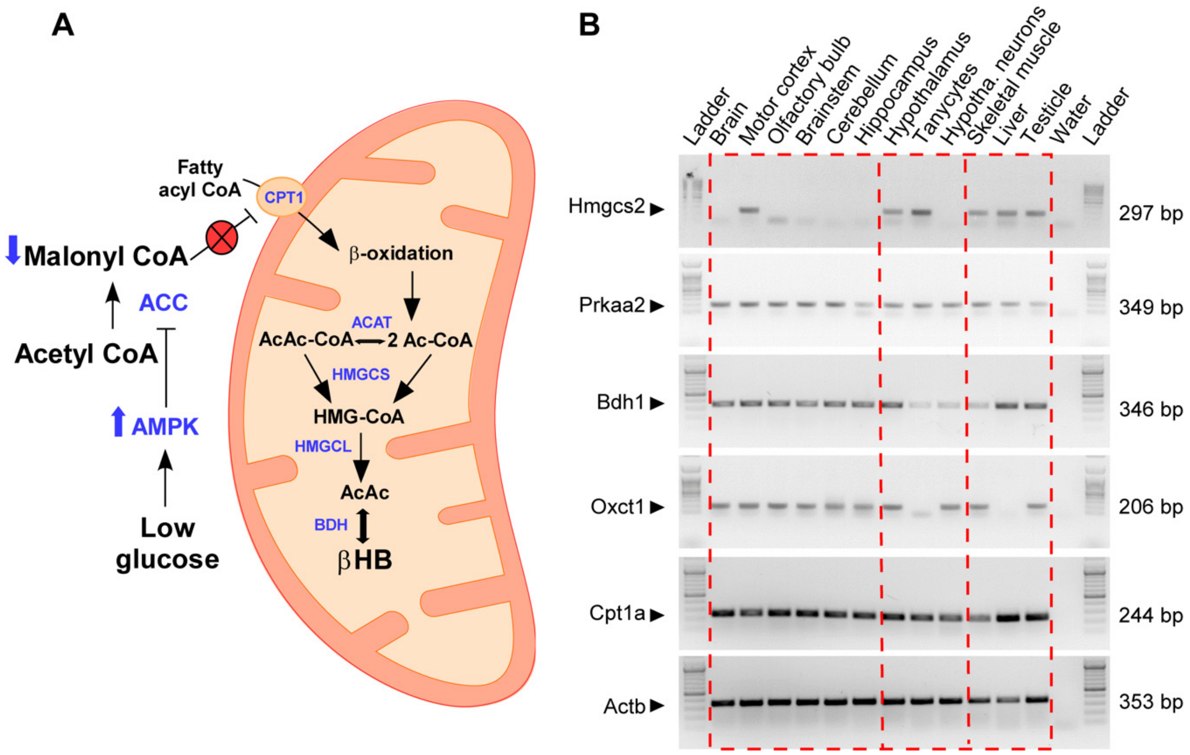

Supp. Fig 1: Tanycytes express critical factors involved in βHB synthesis. **A**. Scheme showing the participation of critical proteins in mitochondrial fatty acids incorporation and metabolism for the synthesis of βHB in low glucose. CPT1: Carnitine palmitoyl transferase 1; ACAT: Acetyl-CoA acetyltransferase; HMGCS: 3-hydroxy-3-methylglutaryl-CoA synthase; HMGCL: 3-hydroxy-3-methylglutaryl-CoA lyase; AcAc-CoA: acetoacetyl-CoA; AcAc: Acetoacetate; ACC: acetyl-CoA carboxylase; BDH: β-Hydroxybutyrate Dehydrogenase 1. **B**. RT-PCR analysis of mRNA expression from cerebral and peripheral tissues and primary cells. Motor cortex (lane 2), hypothalamus (lane 7), primary culture of tanycytes (lane 8), skeletal muscle (lane 10), liver (lane 11) and testicles (lane 12) express significant mRNA amount for Hmgcs2 (297 bp) and Bdh1 (346 bp), two key enzymes to catalyze ketogenesis from acetyl-CoA. Tanycytes also express Prkaa2 (349 bp) and Cpt1a (244 bp) whereas liver (lane 11) and tanycytes do not express detectable levels of 3-oxoacid CoA-transferase 1 (Oxct1), involved in βHB catabolism. β-actin mRNA (Actb, 353 bp) was used as a loading control. Square highlights the positions of the samples.

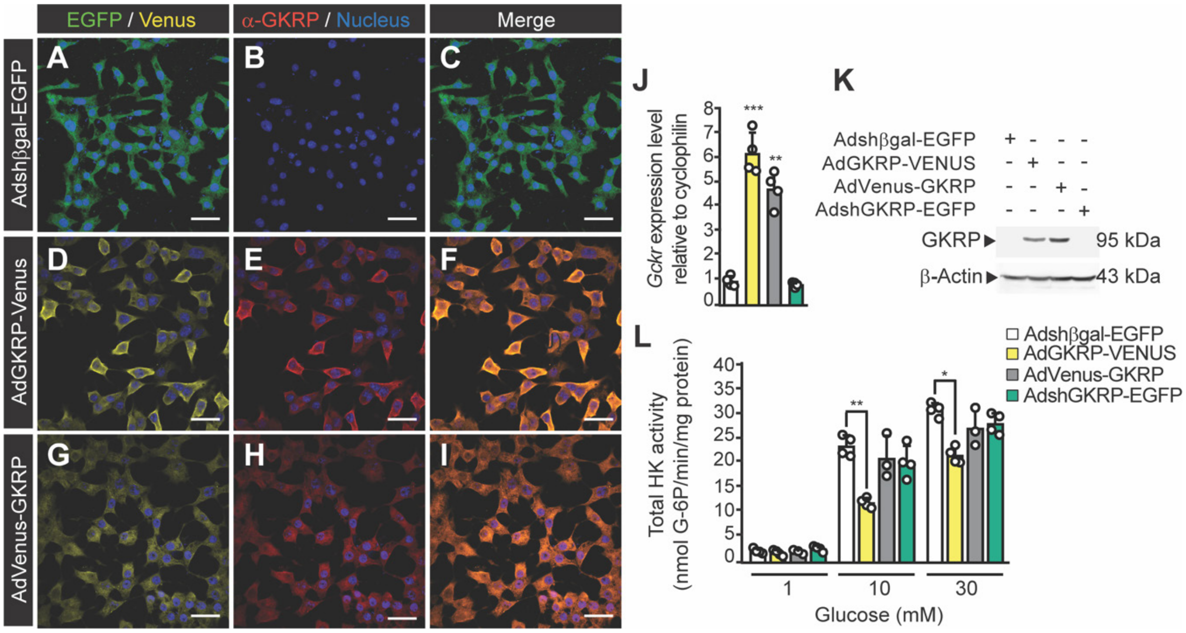

Supp. Fig 2: Functional validation of the viruses produced to overexpress GKRP. 832-13 cells were transduced with 5 x 10^7 IFU/mL of each virus for 72 h and then processed for confocal microscopy (**A-I**), RNA extraction, and *Gckr* quantification by RT-qPCR (**J**) and Western blot (**K**). **L**. Hexokinase (HK) activity at different glucose concentrations in 832-13 cells transduced with each adenovirus. ** $p < 0.01$, *** $p < 0.001$. n = 6

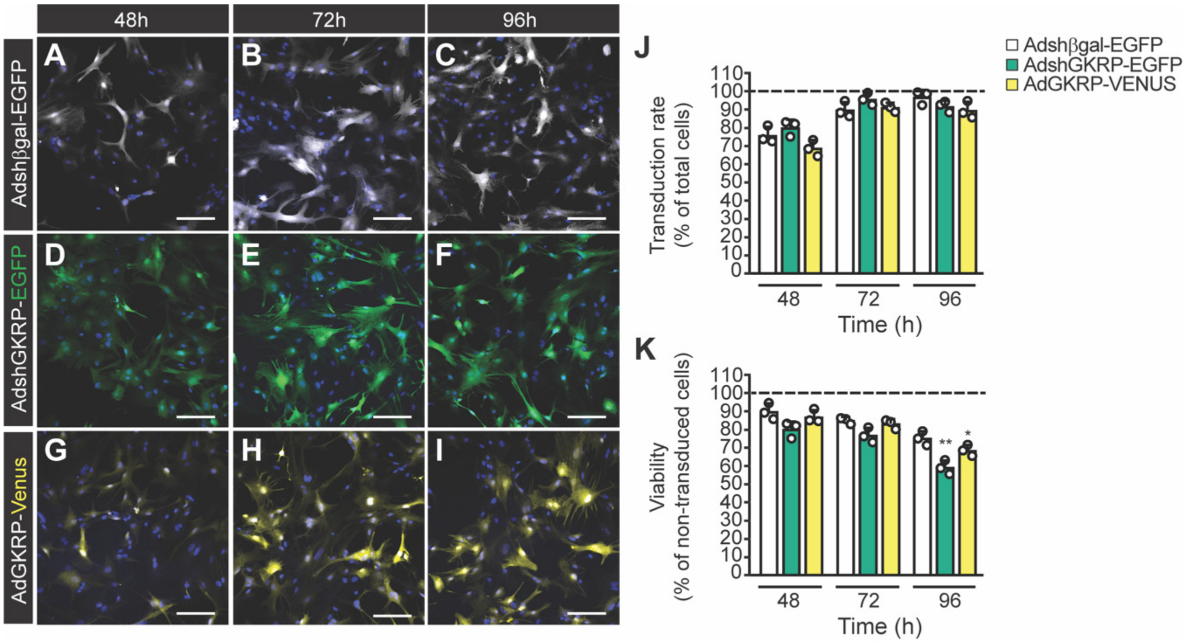

Supp. Fig 3: Optimization of adenovirus transduction. **A-K**: Evaluation of transduction rate by immunocytochemistry (**A-J,** n=3) and viability through trypan blue staining (**K,** n = 3) of tanycyte cultures after 48, 72, and 96 h of transduction with Adshβgal-EGFP, AdshGKRP-EGFP, and AdGKRP-Venus.

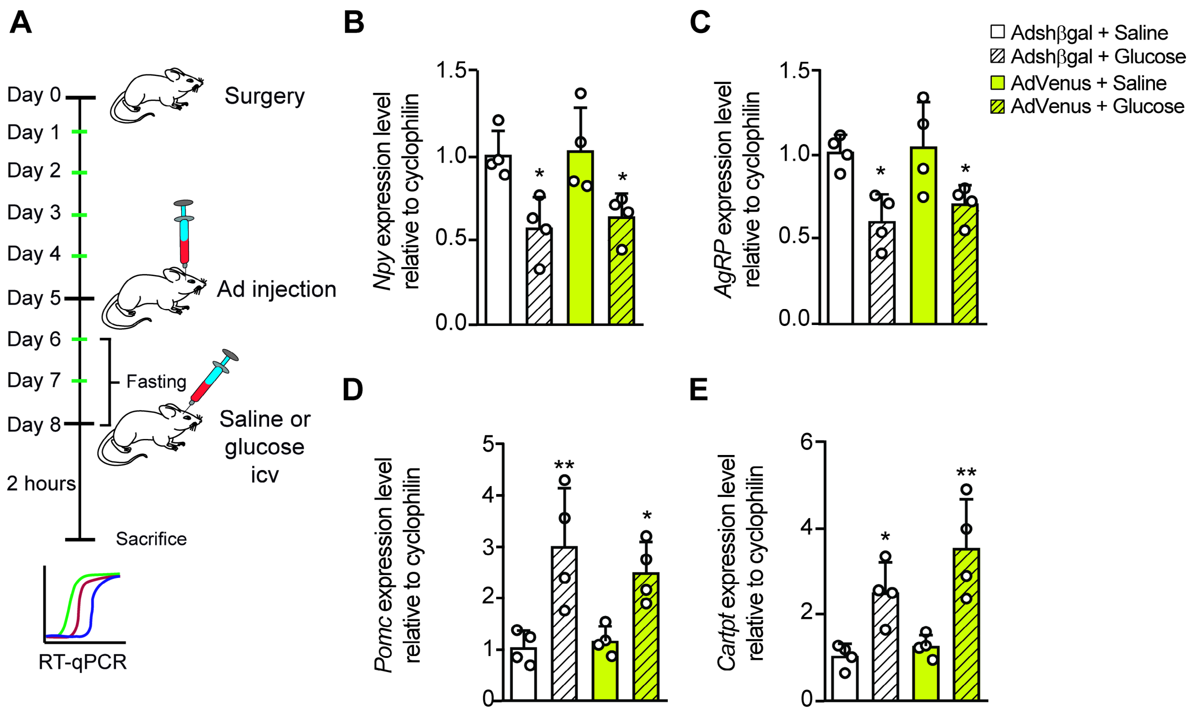

Supp. Fig 4: Comparison of glucose response in animals transduced with Adshβgal-EGFP (Adshβgal) and Ad-Venus. A. Experimental scheme of the protocol used to determine the expression profile of hypothalamic neuropeptides in response to glucose. After 3 days of viral transduction, the animals were icv-injected with 10 µL of 0.9% NaCl or 10 µL of 50 mM glucose to reach hyperglycorrhachia.–NPY (**B**), AgRP (**C**), POMC (**D**), and CART (**E**) mRNA expression was determined in animals transduced with Adshβgal (white bars) and Ad-Venus (yellow bars). We found an indistinguishable response to glucose by using each control adenovirus either by evaluating the expression of orexigenic and anorexigenic neuropeptides. n = 4 per condition. * $p < 0.05$, ** $p < 0.01$.
